# Regulated IRE1α-dependent decay (RIDD)-mediated reprograming of lipid metabolism in cancer

**DOI:** 10.1038/s41467-022-30159-0

**Published:** 2022-05-06

**Authors:** Aitor Almanza, Katarzyna Mnich, Arnaud Blomme, Claire M. Robinson, Giovanny Rodriguez-Blanco, Sylwia Kierszniowska, Eoghan P. McGrath, Matthieu Le Gallo, Eleftherios Pilalis, Johannes V. Swinnen, Aristotelis Chatziioannou, Eric Chevet, Adrienne M. Gorman, Afshin Samali

**Affiliations:** 1 Apoptosis Research Centre, National University of Ireland, Galway, H91 W2TY Ireland; 2 School of Biological and Chemical Sciences, National University of Ireland, Galway, H91 W2TY Ireland; 3 CRUK Beatson Institute, Garscube Estate, Switchback Road, Glasgow, G61 1BD UK; 4 metaSysX GmbH, 14476 PotsdGarscube Estateam-Golm, Germany; 5 Inserm U1242, University of Rennes, Rennes, France; 6 Centre de lutte contre le cancer Eugène Marquis, Rennes, France; 7 e-NIOS Applications PC, 25 Alexandros Pantou str., 17671 Kallithea, Greece; 8 Department of Oncology, Laboratory of Lipid Metabolism and Cancer, KU Leuven Cancer Institute, Leuven, Belgium; 9 Center of Systems Biology, Biomedical Research Foundation of the Academy of Athens, 4 Soranou Ephessiou str, 11527 Athens, GR Greece

**Keywords:** RNA, Cancer metabolism

## Abstract

IRE1α is constitutively active in several cancers and can contribute to cancer progression. Activated IRE1α cleaves *XBP1* mRNA, a key step in production of the transcription factor XBP1s. In addition, IRE1α cleaves select mRNAs through regulated IRE1α-dependent decay (RIDD). Accumulating evidence implicates IRE1α in the regulation of lipid metabolism. However, the roles of XBP1s and RIDD in this process remain ill-defined. In this study, transcriptome and lipidome profiling of triple negative breast cancer cells subjected to pharmacological inhibition of IRE1α reveals changes in lipid metabolism genes associated with accumulation of triacylglycerols (TAGs). We identify *DGAT2* mRNA, encoding the rate-limiting enzyme in TAG biosynthesis, as a RIDD target. Inhibition of IRE1α, leads to DGAT2-dependent accumulation of TAGs in lipid droplets and sensitizes cells to nutritional stress, which is rescued by treatment with the DGAT2 inhibitor PF-06424439. Our results highlight the importance of IRE1α RIDD activity in reprograming cellular lipid metabolism.

## Abstract (web summary)

IRE1α cleaves several mRNAs upon accumulation of misfolded proteins. Here the authors show that active IRE1α cleaves DGAT2 mRNA encoding the rate-limiting enzyme in the synthesis of triacylglycerols, suggesting a role of IRE1α in reprogramming lipid metabolism in cancer cells.

## Introduction

The endoplasmic reticulum (ER) is the largest organelle in the cell and is involved in multiple fundamental biological processes including protein folding and secretion, Ca^2+^ storage and lipid homeostasis. In response to an accumulation of misfolded proteins in the ER lumen, an adaptive-response, named the unfolded protein response (UPR), is activated to restore ER homeostasis. The ER transmembrane protein inositol-requiring enzyme 1 α (hereafter referred to as IRE1α, also known as *ERN1*) is the most conserved UPR signal transducer^1^. IRE1α is a type I transmembrane protein composed of an N-terminal ER luminal domain that senses protein misfolding and a C-terminal cytosolic portion containing kinase and endoribonuclease (RNase) domains^2^. ER stress triggers IRE1α dimerization/oligomerization and *trans*-autophosphorylation, leading to an activating conformational change of the RNase domain. Activated IRE1α RNase cleaves X-box binding protein 1 (*XBP1*) mRNA at two consensus sites, leading to the excision of a 26 nucleotide intron^3,4^. Subsequent ligation of *XBP1* fragments by the tRNA ligase RTCB^5–7^ results in the translation of a transcription factor, spliced XBP1 (XBP1s)^5^. IRE1α RNase also cleaves other select mRNAs via a specific process called regulated IRE1-dependent decay (RIDD) leading to their exonuclease-mediated degradation^3^. Thus, stress-activated IRE1α orchestrates a complex multilayer transcriptional, post-transcriptional and post-translational biological response ultimately aiming at mitigating ER stress^8^. While XBP1s is recognized as the main factor regulating the expression of the adaptive and pro-survival factors required to restore ER homeostasis^9^, the functional significance of RIDD remains largely unexplored and has been associated with both pro-survival and pro-death functions depending on the biological context^10^.

Consistent with its pivotal role in governing ER biology, alterations in IRE1α signaling have been linked to a wide range of human disorders including cancer^8^. A large body of evidence suggests a role for IRE1α in oncogenesis and tumor progression^11^. Aberrant constitutive IRE1α activity has been reported in many cancer types, including the aggressive triple negative breast cancer (TNBC)^12–14^. Recent work from our lab and others has shown that blocking IRE1α RNase activity in TNBC can enhance the effectiveness of current chemotherapies and delay post-therapy tumor relapse^13–15^. However, while these reports establish the IRE1α/XBP1s signaling axis as a major player in TNBC pathology, the significance of RIDD activity to this disease remains to be determined. Interestingly, in some cancers, for instance glioblastoma, RIDD and XBP1s are associated with different and sometimes opposite functions^16^.

Accumulating evidence suggests a role for IRE1α in the regulation of lipid metabolism, especially in triacylglycerol (TAG) metabolism and storage, fatty acid (FA) oxidation and membrane phospholipid production^17–19^. Importantly, metabolic reprograming is a hallmark of many cancer types^20^. In TNBC, enhanced lipid metabolism can support tumor progression^21^. However, the role of IRE1α in the regulation of TNBC lipid metabolism remains largely uncharacterized.

In this study, we employed unbiased lipidomic and transcriptomic approaches to investigate the contribution of IRE1α signaling to lipid metabolism in TNBC. The integration of these data enabled us to identify TAG metabolism as the most altered lipid biosynthetic pathway upon IRE1α RNase inhibition. We generated a list of potential IRE1α target genes that are involved in TAG metabolism. From these genes, we identified *DGAT2* as a RIDD substrate and uncovered the importance of DGAT2-mediated regulation of TAG metabolism in TNBC, thus linking IRE1α activity to TAG biosynthesis in cancer cells. Furthermore, we demonstrated that IRE1α inhibition sensitized cancer cells to starvation through increased DGAT2 activity. These findings suggest a key role for RIDD in cancer and demonstrate the importance of IRE1α in the regulation of cellular lipid metabolism and adaptation to stress.

## Results

### Inhibition of IRE1α RNase activity alters cellular lipid composition

**Fig. 1:**
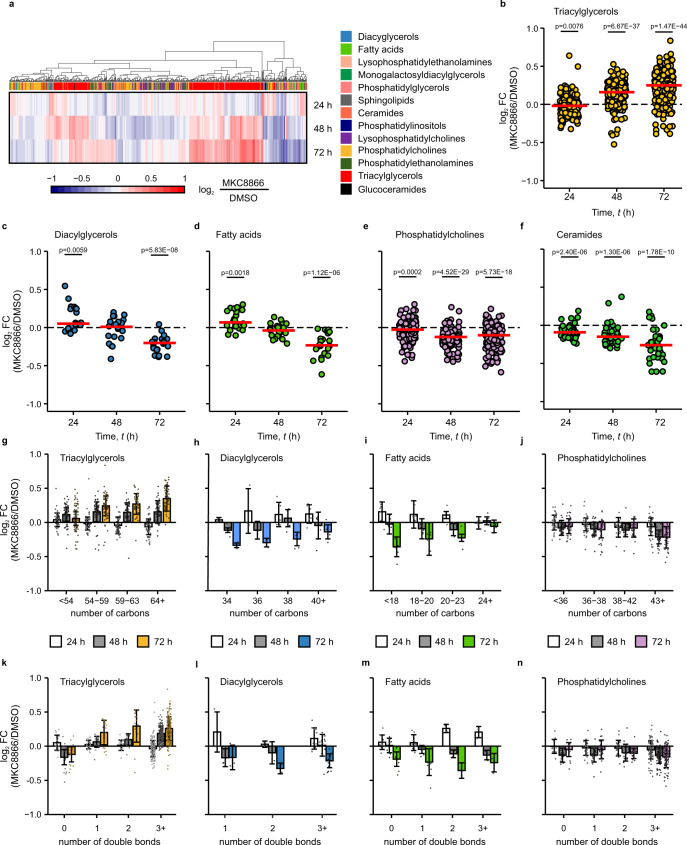
Lipidomic profiling reveals changes in TAG metabolism upon inhibition of IRE1α. **a**–**n** MDA-MB-231 cells were treated with 20 µM MKC8866 or vehicle (DMSO) for 24, 48, and 72 h after which their lipidomic landscape was profiled by ultraperformance liquid chromatography tandem mass spectrometry (UPLC–MS) (*n* = 5 biologically independent experiments). **a** Heatmap showing the log_2_-fold change (log_2_ FC) between the MKC8866 and DMSO groups for each measured lipid species. Red indicates increased abundance while blue indicates those with lower abundance upon MKC8866 treatment. Lipid class is indicated in column color tags. **b**–**f** Scatter plots showing the average log_2_ FC (MKC8866/DMSO) for levels of **b** TAGs (*n* = 242), **c** DAGs (*n* = 20), **d** FAs (*n* = 22), **e** PCs (*n* = 145), and **f** ceramides (*n* = 41) species at the indicated time points. **g**–**n** Bar plots displaying log_2_ FC (MKC8866/DMSO) for levels of **g**, **k** TAG, **h**, **l** DAG, **i**, **m** FA and **j**, **n** PC lipid groups separated by **g**–**j** chain length and **k**–**n** unsaturation status. Individual dots representing the average log_2_ FC for each lipid species. Data are presented as mean log_2_ FC values ± s.d. Statistical comparisons performed with unpaired one-sample *t*-test to determine whether the population mean is different from 0. *p* values indicated on the graph were considered significant if *p* < 0.05. Source data are provided as a Source Data file.

The impact of IRE1α RNase activity on lipid metabolism was assessed in MDA-MB-231 cells, which exhibit high basal IRE1α activity^13^. MDA-MB-231 cells were treated with the IRE1α RNase inhibitor MKC8866^22^ for up to 72 h (Supplementary Fig. 1a) and their lipid profile was analyzed by liquid chromatography coupled with mass spectrometry (LC–MS). Inhibition of IRE1α gradually altered intracellular lipid content and composition over time (Fig. 1a–f, Supplementary Fig. 1b–g). After 48 and 72 h of treatment with MKC8866, cells exhibited higher levels of most TAG species in comparison to control cells (Fig. 1b). Concomitantly, there were reduced levels of diacylglycerols (DAGs) (Fig. 1c), free FAs (Fig. 1d), phosphatidylcholines (PCs) (Fig. 1e), and ceramides (Fig. 1f). A detailed analysis of the different lipid classes (Supplementary Fig. 2a–c) revealed that IRE1α inhibition preferentially increased the abundance of mono- and polyunsaturated long chain TAG species (Fig. 1g, k). By contrast, MKC8866-treated cells displayed decreased levels of saturated TAGs, while the levels of TAGs with less than 54 carbons remained mostly unaffected by MKC8866 treatment. The concurrent decrease in abundance of DAGs (Fig. 1h, l), 18–23 carbon monounsaturated FAs (MUFAs) and polyunsaturated FAs (PUFAs) (Fig. 1i, m), combined with the reduced levels of polyunsaturated and elongated phosphatidylcholines (Fig. 1j, n) suggested that MKC8866 caused a shift in lipid metabolism, favouring increased TAG synthesis^23^.

### IRE1α modulates the expression of genes involved in TAG metabolism

**Fig. 2:**
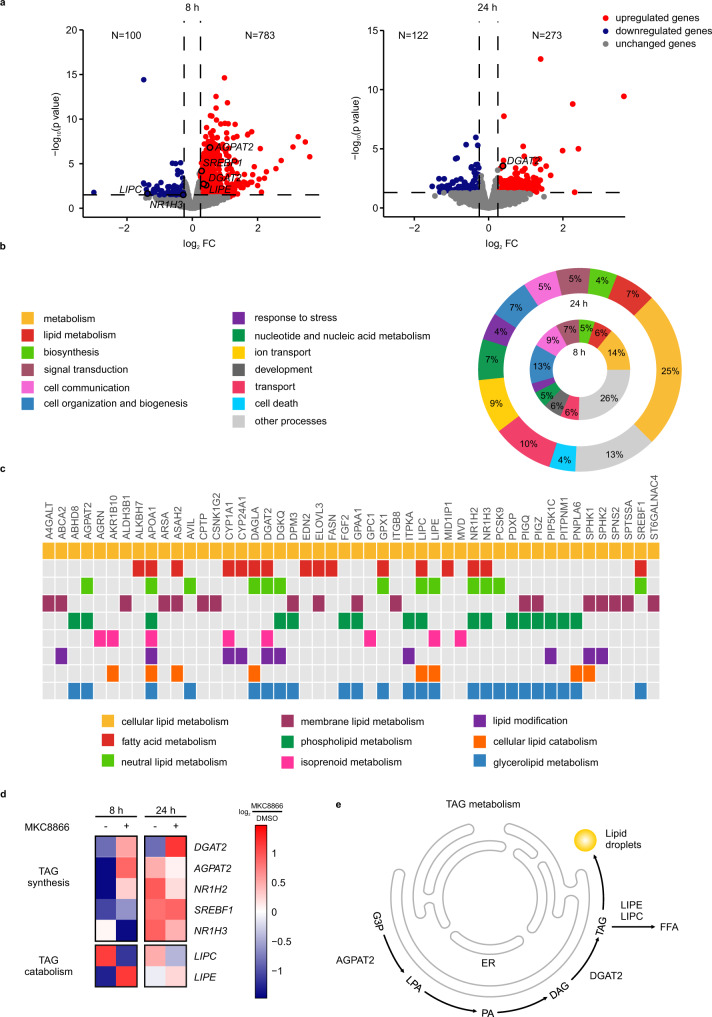
IRE1α signaling modulates expression of genes involved in TAG metabolism. **a**–**c** MDA-MB-231 cells were treated with 20 µM MKC8866 or DMSO for 8 and 24 h after which RNA was extracted and RNA sequencing analysis performed (*n* = 3 biologically independent experiments). Pairwise comparison MKC8866/DMSO was performed with edgeR for identification of differentially expressed genes (DEGs) at each time point (no multiple comparison adjustments were performed). **a** Volcano plots showing fold changes and −log_10_(*p*-value) for genes differentially expressed between MKC8866 and DMSO treated cells at 8 and 24 h. Blue indicates downregulated genes and red indicates upregulated genes upon MKC8866 treatment (*p* < 0.05). **b** Summary of a functional analysis of DEGs at 8 and 24 h time points. Overrepresented Gene Ontology (GO) terms were summarized into broader biological groups based on semantic similarity with cateGOrizer to facilitate biological interpretation. **c** Genes termed significantly changing at either of the two time points and annotated to lipid metabolism on the GO database. **d** Heatmap displaying average expression levels for DEG annotated to TAG metabolism in the GO database. The expression for each gene (row) was centered and scaled so the mean expression is zero and standard deviation is one. **e** Schematic representation of TAG metabolism, showing TAG metabolism-regulating genes whose expression is altered by IRE1α. G3P glycerol-3-phosphate, LPA lysophosphatidic acid, PA phosphatidic acid, DAG diacylglycerol, TAG triacylglycerol, FFA free fatty acid. Source data are provided as a Source Data file.

To gain further insights into the molecular mechanisms underlying lipid remodeling upon IRE1α inhibition, we carried out a transcriptomic profiling of MDA-MB-231 cells treated with or without MKC8866 for 8 and 24 h using RNA-sequencing. MKC8866 inhibition of IRE1α RNase activity was confirmed by changes in the expression levels of *XBP1s* mRNA, and of representative genes that are downstream of XBP1s: DNAJ heat shock protein family member B9 (*DNAJB9*), homocysteine-responsive ER-resident protein with ubiquitin like domain 1 (*HERPUD1*, also known as *HERP*), and *DNAJC3* (also known as *P58IPK*) (Supplementary Fig. 3a–d). Pairwise comparison between MKC8866 and DMSO-treated samples identified 883 differentially expressed genes (DEGs) at 8 h and 395 DEGs at 24 h (Fig. 2a). Functional analysis was performed with BioInfoMiner (Supplementary Table 1)^24^ and results were summarized into broader biological groups based on semantic similarity with cateGOrizer^25^ (Fig. 2b). IRE1α inhibition resulted in marked changes in the expression of numerous genes involved in lipid metabolism (Fig. 2b, c). In agreement with the lipidomics data (Fig. 1), we observed that inhibition of IRE1α led to the differential expression of several genes involved in TAG metabolism (Fig. 2d, e). Among these, *SREBF1, DGAT2*, and *LIPE* were all upregulated following short-term exposure to MKC8866 treatment, thus identifying these genes as potential IRE1α RIDD substrates.

### *DGAT2* is a RIDD target

**Fig. 3:**
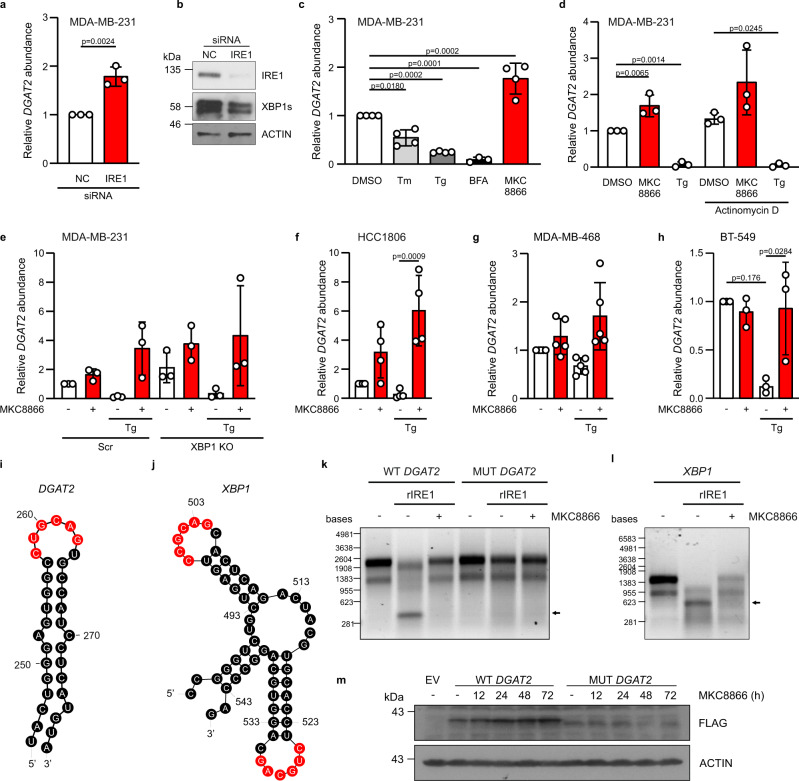
*DGAT2* is an IRE1α RIDD substrate. **a**–**d** MDA-MB-231 cells were either **a**, **b** transfected with non-coding (nc) and IRE1 siRNA for 72 h (*n* = 3 biologically independent experiments), **c** treated with Tunicamycin (Tm, 5 µg/ml), Thapsigargin (Tg, 0.25 µM), Brefeldin A (BFA, 0.5 µg/ml), MKC8866 (20 µM) for 24 h (*n* = 4 biologically independent experiments) or **d** pre-treated with actinomycin D (2 µg/ml) for 2 h followed by incubation of cells with Tg (0.25 µM) and MKC8866 (20 µM) for 8 h (*n* = 3 biologically independent experiments). **e** MDA-MB-231 XBP1 knockout (KO) cells were treated with DMSO or 20 µM MKC8866 in the presence or absence of 1 µM Tg (*n* = 3 biologically independent experiments). RT-qPCR quantification of *DGAT2* transcript levels relative to **a**, **c**, **d** *MRPLP19* and **e** *ACTB* and normalized to control at 0 h time point. **b** Immunoblotting of total IRE1α, XBP1s and ACTIN. **f**–**h** Expression of *DGAT2* relative to *MRPL19* in **f** HCC1806 (*n* = 4 biologically independent experiments), **g** MDA-MB-468 (*n* = 5 biologically independent experiments) and **h** BT-549 cells (*n* = 3 biologically independent experiments) treated with DMSO or 20 µM MKC8866 in the presence or absence of 1 µM Tg for 24 h. **i**, **j** *RNAfold* prediction of secondary structure of mRNA fragments of **i** *DGAT2* and **j** *XBP1* mRNA. IRE1α consensus cleavage sequences are highlighted in red. **k**, **l** In vitro transcribed **k** wild type (WT) *DGAT2*, c.260G>A *DGAT2* mutant (MUT *DGAT2*)and **l** *XBP1* mRNAs were incubated with recombinant hIRE1α in the presence or absence of 20 μM MKC8866. Arrows indicate cleavage products (*n* = 4 biologically independent experiments). **m** MDA-MB-231 cells stably expressing empty vector (EV), FLAG-tagged wild type *DGAT2* (WT *DGAT2*) or guanine 260 to adenine mutated FLAG-tagged *DGAT2* (MUT *DGAT2*) were treated with 20 μM MKC8866 for indicated time points. Representative immunoblots of FLAG and ACTIN for three biologically independent experiments. Indicated *p* values, based on **a** comparison of the two groups using an unpaired two-tailed *t* test or **c**–**h** one-way ANOVA with Bonferroni’s multiple comparisons post hoc tests. Values with *p* < 0.05 are considered statistically significant. Data are presented as mean values ± s.d. Source data are provided as a Source Data file.

Among the potential IRE1α targets involved in TAG metabolism, we focused our attention on *DGAT2*, as this gene encodes an ER membrane-localized enzyme that catalyzes the terminal, rate-limiting step in TAG synthesis^26^. In agreement with the transcriptomic data, RT-qPCR analysis revealed increased *DGAT2* mRNA levels in MDA-MB-231 cells depleted of IRE1α (Fig. 3a, b) or treated with MKC8866 (Fig. 3c). By contrast, IRE1α activation, using classical ER stress inducers tunicamycin (Tm), thapsigargin (Tg) and brefeldin A (BFA), decreased *DGAT2* mRNA expression in these cells (Fig. 3c). To test whether the MKC8866-dependent increase in *DGAT2* expression resulted from increased gene transcription or mRNA post-transcriptional regulation, we treated MDA-MB-231 cells with Tg or MKC8866 in the presence or absence of the transcription inhibitor actinomycin D. In both cases, Tg reduced *DGAT2* levels while MKC8866 increased *DGAT2* expression independently of transcription (Fig. 3d). As a positive control, ER stress induction of *HSPA5* mRNA was blocked by actinomycin D treatment (Supplementary Fig. 3e). A similar pattern was observed in *XBP1* knockout MDA-MB-231 cells (Supplementary Fig. 3f), where lack of XBP1s did not affect the pattern of changes in *DGAT2* transcript levels upon inhibition or activation of IRE1α activity using MKC8866 or Tg, respectively (Fig. 3e).

We further examined changes in expression of *DGAT2* in additional TNBC cell lines HCC1806, MDA-MB-468, and BT-549 following treatment with MKC8866 and Tg. The cells exhibited varying changes in *DGAT2* mRNA levels in response to treatment with MKC8866 alone (Fig. 3f–h), which reflects their basal IRE1α activities (Supplementary Fig. 3g, h)^13^. However, in all of the cell lines tested changes in *DGAT2* mRNA levels followed a similar pattern in response to activation of IRE1α (treatment with Tg) and this effect was reversed by MKC8866 co-treatment (Fig. 3f–h). Treatment of MCF10A cells, a non-transformed, non-tumorigenic breast epithelial cell line, which do not display constitutive IRE1α RNase activity^13^, with MKC8866, did not alter *DGAT2* mRNA expression (Supplementary Fig. 3i).

In silico analysis of the *DGAT2* mRNA secondary structures using the *RNAfold* Web server^27^ predicted that *DGAT2* contains IRE1α consensus sequence 5′-CUG↓CAG-3′ at the position 258–263 of the coding sequence, located within a stem-loop secondary structure similar to the one observed in *XBP1* (Fig. 3i, j). Therefore, we tested the ability of IRE1α to directly cleave *DGAT2* mRNA (CCDS21477.1). In vitro processing of *DGAT2* and *XBP1* transcripts by recombinant hIRE1α (465–977) resulted in the appearance of a low molecular weight cleavage fragment, detected by agarose gel electrophoresis (Fig. 3k, l). Inhibition of IRE1α or introduction of a single point mutation (c.260G>A), in the *DGAT2* transcript prevented IRE1α cleavage of *DGAT2* mRNA (Fig. 3k). Next, we generated MDA-MB-231 cells stably overexpressing either the FLAG-tagged wild type *DGAT2* (WT *DGAT2*) or the RIDD resistant (c.260G>A) *DGAT2* (MUT *DGAT2*) constructs. Treatment of these cells with 20 µM MKC8866 increased WT DGAT2 but not MUT DGAT2 protein levels for up to 72 h (Fig. 3m). Collectively, these results establish *DGAT2* as a bona fide RIDD target in TNBC cells.

### DGAT2 mediates incorporation of TAGs into LDs upon IRE1α inhibition

**Fig. 4:**
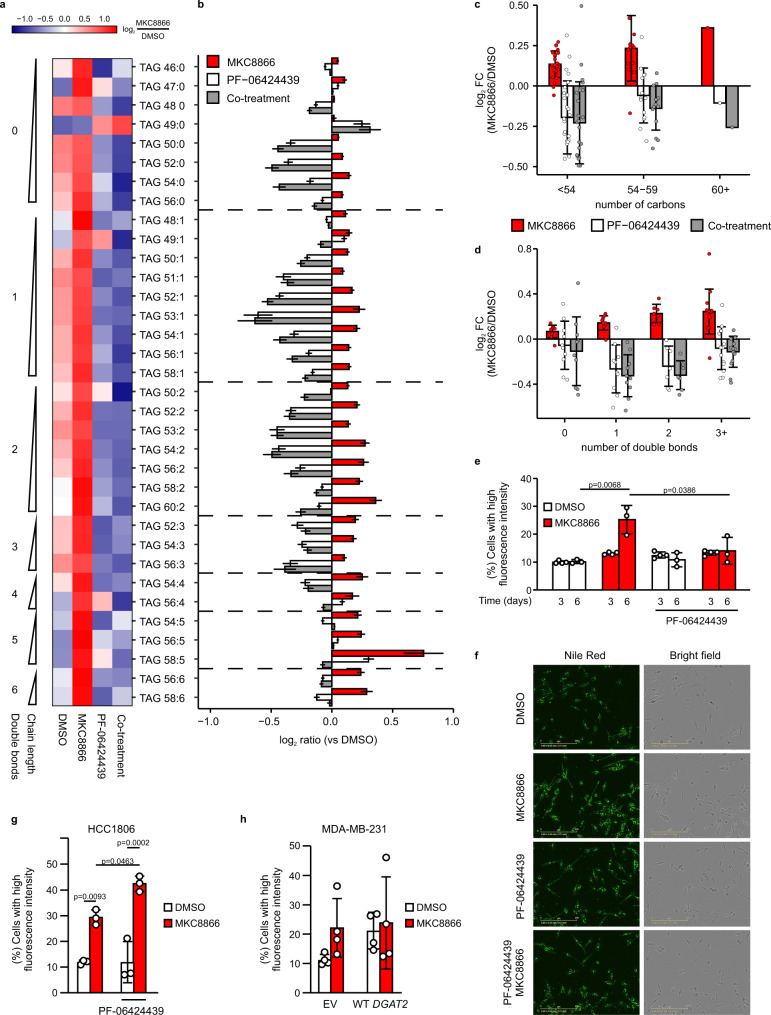
Inhibition of DGAT2 reverses IRE1α-mediated effect on TAG accumulation. **a**–**f** MDA-MB-231 cells, **g** HCC1806 (*n* = 3 biologically independent experiments) and **h** MDA-MB-231 cells stably expressing EV and WT *DGAT2* (*n* = 4 biologically independent experiments) were treated with 20 µM MKC8866 or vehicle for **a**–**d** 3 days, **e** 3 days (*n* = 4 biologically independent experiments) and 6 days (*n* = 3 biologically independent experiments) or **f**–**h** 6 days in the presence or absence of 2 µM PF-06424439. **a**–**d** Metabolites were extracted and their levels were quantified by LC-MS**. a** Heatmap showing the average abundance for all measured TAG species (*n* = 34) across the different treatments. Expression across each row was centered and scaled so the mean expression is zero and standard deviation is one. **b** Bar charts showing mean of log_2_ FC versus vehicle-treated cells (DMSO) ± s.e.m. estimated by propagation of error for each treatment and individual TAG species. **c** and **d** Graphs displaying log_2_ FC versus vehicle-treated cells (DMSO) for each treatment for all TAG species grouped by total number of **c** carbon atoms and **d** double bonds. Bars indicate mean log_2_ FC for lipid species within each group ± s.d. **e**–**h** Cells were stained with Nile red to visualize and quantify lipid droplets. **e**, **g**, **h** Percentage of cells with a high LD content. **f** Representative images of MDA-MB-231 cells stained with Nile red after 6 days of treatment (*n* = 3 biologically independent experiments). Scale bar = 200 µm. Indicated *p* values based on one-way ANOVA with Bonferroni’s multiple comparisons post hoc tests. Values with *p* < 0.05 are considered statistically significant. Data are presented as mean values ± s.d. Source data are provided as a Source Data file.

To demonstrate the functional relevance of DGAT2 in the MKC8866-mediated accumulation of TAGs, we treated MDA-MB-231 cells with MKC8866 in the presence or absence of the DGAT2 inhibitor PF-06424439^27^ and measured TAG levels by LC–MS. MKC8866 treatment resulted in increased levels of several TAG species, and this effect was abolished by co-treatment with PF-06424439 (Fig. 4a, b). IRE1α inhibition led to a preferential accumulation of long-chain PUFAs into TAGs (Fig. 4c, d). DGAT2 inhibition suppressed accumulation of all TAG species irrespective of carbon content or degree of unsaturation (Fig. 4c, d). MKC8866 treatment also resulted in an accumulation of lipid droplets (LDs), the main storage compartments of TAGs in cells^28^, as evidenced by increased uptake of Nile red in MKC8866-treated MDA-MB-231 and HCC1806 cells after 6 days (Fig. 4e-g). This accumulation of LDs was inhibited by treatment with PF-06424439 in MDA-MB-231 cells (Fig. 4e, f). In HCC1806 cells, co-treatment with MKC8866 and PF-06424439 produced even higher LD formation, suggesting involvement of DGAT1 in converting DAGs into TAGs (Fig. 4g). Indeed, we confirmed this using a DGAT1 inhibitor, PF-04620110, which when combined with MKC8866 and PF-06424439 reduced LD formation in these cells (Supplementary Fig. 2d). Treatment of MCF10A cells with MKC8866 for 6 days did not result in LD accumulation in agreement of lack of *DGAT2* mRNA regulation by IRE1α (Supplementary Fig. 2e). To further confirm the involvement of DGAT2 in the observed accumulation of TAGs in LDs, we used MDA-MB-231 cells overexpressing WT *DGAT2*. Similar to parental MDA-MB-231 cells (Fig. 4e), treatment of empty vector (EV) control cells with MKC8866 increased LD levels (Fig. 4h). In contrast, cells overexpressing WT *DGAT2* also exhibited higher levels of LDs when compared to cells expressing EV and treatment with MKC8866 did not result in a further increase in LD levels (Fig. 4h).

### Accumulation of LDs sensitizes cells to starvation upon IRE1α inhibition

**Fig. 5:**
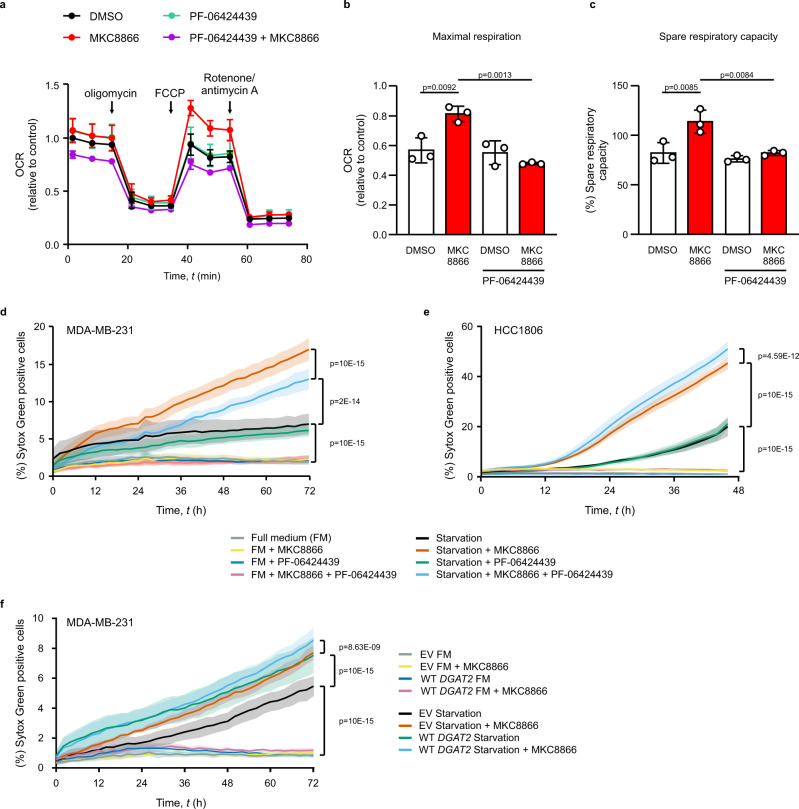
MKC8866 sensitizes cells to starvation through regulation of DGAT2. **a**–**c** MDA-MB-231 cells were treated with vehicle or 20 μM MKC8866 in the presence or absence of 2 µM PF-06424439 for 6 days (*n* = 3 biologically independent experiments). **a**–**c** Seahorse extracellular flux analysis of oxygen consumption rate (OCR) following the indicated injections normalized to vehicle. Data plotted to demonstrate the changes in mitochondrial **b** maximal respiration and **c** spare respiratory capacity. Indicated *p* values based on comparison of multiple groups using one-way ANOVA with Bonferroni’s multiple comparisons post hoc tests. Values with *p* < 0.05 are considered statistically significant. Data are presented as mean values ± s.d. **d** MDA-MB-231 cells (*n* = 4 biologically independent experiments), **e** HCC1806 (*n* = 4 biologically independent experiments) and **f** MDA-MB-231 cells stably expressing EV or WT *DGAT2* (*n* = 3 biologically independent experiments) were treated with vehicle or 20 μM MKC8866 in the presence or absence of 2 μM PF-06424439. After 6 days, culture medium was replaced with a complete medium or Hanks’ balanced salt solution. Kinetics of cell death for up to 72 h was expressed as the percentage of cells that were Sytox Green positive. Indicated *p* values based on two-way ANOVA with Bonferroni’s multiple comparisons post hoc tests. Values with *p* < 0.05 are considered statistically significant. Data are presented as mean values ± s.e.m. Source data are provided as a Source Data file.

TNBC cells exhibit reduced LD abundance compared with normal breast epithelial cells and reliance on fatty acid oxidation (FAO)^29^. We performed Mito Stress Tests using the Seahorse extracellular flux analyzer to quantify the mitochondrial oxygen consumption rate (OCR) in MDA-MB-231 and HCC1806 cells incubated with MKC8866 for 6 days in the presence or absence of PF-06424439. MKC8866 treatment did not affect basal OCR, ATP production or proton leak in MDA-MB-231 (Supplementary Fig. 4a–d) nor did it affect respiratory capacity in HCC1806 (Supplementary Fig. 4e). By contrast, IRE1α inhibition significantly increased both maximal and spare mitochondrial respiratory capacities (SRC) in MDA-MB-231 cells when compared to DMSO-treated cells, an effect that was reversed by DGAT2 inhibition (Fig. 5a–c). Rapidly proliferating cancer cells have low SRC because they utilize mitochondrial reserves to meet extra energy requirements during mitosis^30^. Long-term (6 days) inhibition of IRE1α significantly inhibited MDA-MB-231 cell proliferation^13^ (Supplementary Fig. 4f). However, this did not rely on DGAT2, since PF-06424439 had no effect (Supplementary Fig. 4f).

Higher mitochondrial respiratory capacity may provide an additional advantage for cells to cope with metabolic stress, such as limited availability of nutrients in the tumor microenvironment. To assess whether LD accumulation following IRE1α inhibition can impact cancer cell survival under metabolic stress conditions, MDA-MB-231 were cultured with MKC8866 for 6 days in the presence or absence of PF-06424439 followed by 3 days of starvation in Hanks’ balanced salt solution (HBSS) (Fig. 5d), 2% serum (Supplementary Fig. 4g) or glucose deprivation (Supplementary Fig. 4h). We monitored the effects on cell death by Sytox Green uptake. MDA-MB-231 cells were relatively resistant to death in conditions of nutrient deprivation (Fig. 5d, Supplementary Fig. 4g, h). Treatment with MKC8866 significantly sensitized the cells to death upon starvation, and this effect was reversed by inhibition of DGAT2 (Fig. 5d). In HCC1806 cells, treatment with MKC8866 also significantly sensitized cells to starvation, and inhibition of DGAT2 sensitized cells even further, in agreement with higher accumulation of LDs in these cells compared with MKC8866-treated cells (Fig. 5e). While MKC8866 itself sensitized MCF10A cells to starvation (most likely due to inhibition of IRE1, which was activated by starvation), inhibition of DGAT2 did not significantly change the response of MCF10A cells to MKC8866 treatment (Supplementary Fig. 4i). To validate that the effect of MKC8866 on cell death was indeed a consequence of increased DGAT2 levels, we examined MDA-MB-231 cells overexpressing WT *DGAT2* and observed increased sensitivity to starvation which was further potentiated by MKC8866 treatment (Fig. 5f). These results suggest that IRE1α-mediated reprograming of lipid metabolism plays a role in the adaptation of cancer cells to nutritional stress.

## Discussion

IRE1α signaling has recently emerged as an important player in cancer biology^31^. In TNBC, the most aggressive breast cancer subtype, IRE1α activity has been linked to tumorigenesis^14^, tumor progression^12,14^, angiogenesis^15^, metastasis^14^, remodeling of tumor microenvironment^13,15^, resistance to anthracycline- or taxane-based chemotherapy^12–14^ and post-therapy tumor relapse^13^. While the IRE1α/XBP1s signaling axis has emerged as an important factor and potential therapeutic target in TNBC, the significance of RIDD activity in this disease remains unexplored. In this study, we identified IRE1α as an important regulator of lipid metabolism in TNBC. Inhibition of IRE1α RNase activity produced a major remodeling of the lipidome of MDA-MB-231 cells, shifting lipid metabolism towards the accumulation of TAGs in lipid droplets. Using a combination of lipidomics, transcriptomics, bioinformatics, RT-qPCR, in vitro cleavage assays and imaging we demonstrated that *DGAT2* mRNA is a RIDD substrate and that its regulation by IRE1α has functional consequences for TAGs synthesis, their incorporation into lipid droplets and adaptation of cancer cells to nutritional stress.

DGAT2 has previously been reported as an IRE1α-responsive gene, yet the molecular mechanisms underlying this regulation remained unclear. Some studies reported *DGAT2* as a XBP1s-induced gene in mice hepatocytes and adipocytes^17,32^. Other studies had already suggested *DGAT2* as a potential RIDD substrate in mice hepatocytes, mainly based on gene expression changes following modulation of IRE1α activity^33–35^. While it is possible that both the XBP1s and RIDD arms of IRE1α contribute to the regulation of *DGAT2*, we demonstrated that IRE1α acts predominantly as a post-transcriptional repressor of *DGAT2* expression through RIDD in MDA-MB-231, HCC1806, BT-549 cells. We showed that *DGAT2* mRNA is directly cleaved by IRE1α RNase at the consensus CUG↓CAG sequence at positions 258–263 within a stem-loop secondary structure. While this manuscript was in preparation, Le Thomas et al. published complementary evidence supporting the *DGAT2* transcript as a RIDD substrate, further confirming our findings^36^.

Several studies support the idea of IRE1α signaling as a suppressor of TAG accumulation. For example, deletion of IRE1α in mouse liver resulted in increased TAG levels and higher LD abundance in hepatocytes^37,38^. Knockout of *Xbp1* in mouse liver caused hypotriglyceridemia, while downregulation of IRE1α restored plasma TAG levels in this context^34^. It is worth noting that ablation of XBP1 has been reported to activate upstream IRE1α and thus potentiate mRNA decay through RIDD^34^. Different mechanisms have been suggested for the IRE1α-mediated regulation of TAGs, such as a decreased FAO through the impairment of the XBP1s/PPARα signaling axis^39^, abrogation of very low-density lipoprotein assembly and TAG secretion^19^ and increased de novo lipogenesis^34,38^. Here, we characterized the molecular nature of the regulation by IRE1α of TAG biosynthesis in TNBC by linking IRE1α RIDD activity to DGAT2 regulation, confirming previous observations linking IRE1α to TAG biosynthesis.

Lipid metabolism reprogramming is an important driver of tumor progression in several cancer types^40^. Thus, the interplay between IRE1α signaling, lipid metabolism, and cancer biology is emerging as an important area for research. To date, only one report demonstrated that IRE1α/XBP1s signaling can directly regulate tumor growth through the upregulation of the lipid desaturase *SCD1* in Myc‐transformed cancer cells^41^. Other studies reported that XBP1s can regulate *PDK1* and *GLUT1* gene expression in MDA-MB-231 cells under hypoxic conditions, however the functional consequences of those changes have not been investigated^12^. Here we demonstrate RIDD activity in reprogramming of lipid metabolism through degradation of *DGAT2* mRNA. Increased levels of DGAT2 (either as a result of IRE1α inhibition or overexpression of *DGAT2*) cause sensitization of MDA-MB-231 and HCC1806 cells to starvation. Our finding that inhibition of IRE1α sensitizes cancer cells to starvation, was surprising as LD accumulation has previously been reported to protect against lipotoxic FAs released upon autophagic breakdown of membranous organelles during starvation^42^. One explanation could be that upon nutrient deprivation, when cancer cells mainly rely on lipids stored in LDs as an energy source^43^, an increased lipolysis of TAGs rich in PUFAs results in lipotoxicity due to peroxidation of PUFAs, a key driver of ferroptosis^9^. Our data support the notion that the effect of MKC8866 on cell viability under starvation is in part due to its effect on DGAT2. Indeed nutrient deprivation causes ER stress, which activates the UPR. If unresolved, this can lead to cell death^44^. The IRE1α arm of the UPR promotes cell survival and adaptation^45^ and thus, inhibiting IRE1α under conditions of prolonged ER stress will sensitize to cell death.

In summary, our data support the growing efforts to provide rationale for targeting IRE1α as a therapeutic strategy to combat TNBC. We provide a mechanistic explanation of metabolic reprogramming in cells with a high IRE1α activity. By showing that IRE1α-mediated cleavage of *DGAT2* plays a key role in the resistance of TNBC cells to nutrient deprivation we highlight the importance of developing IRE1α inhibitors for clinical benefits.

## Methods

### Cell culture and treatments

MDA-MB-231 (ATCC, HTB-26) and HEK293T (ATCC, CRL-3216) cells were cultured in DMEM high glucose (Sigma-Aldrich, D6429) supplemented with 10% fetal bovine serum (FBS) (Sigma-Aldrich, F7524) and 2 mM l-glutamine (Sigma-Aldrich, G7513) at 37 °C, 5% CO_2_ in a humidified incubator. HCC1806 (ATCC, CRL-2335), MDA-MB-468 (ATCC, HTB-132), and BT-549 (ATCC, HTB-122) cells obtained from ATCC were cultured in RPMI-1640 medium (Sigma-Aldrich, R0883) supplemented with 10% FBS and 2 mM l-glutamine. MDA-MB-231 and MCF10A (ATCC, CRL-10317) cells were authenticated by ATCC using short tandem repeat (STR) analysis with 100% match with ATCC reference database profile. MCF10A cells were maintained in DMEM/F-12 (Sigma-Aldrich, D6421) supplemented with 5% horse serum (Gibco, 16050122), 20 ng/ml epidermal growth factor (PeproTech, AF-100-15), 0.5 μg/ml hydrocortisone (Sigma-Aldrich, H0888), 100 ng/ml cholera toxin (Sigma-Aldrich, C8052), and 10 μg/ml insulin (Sigma-Aldrich, I1882). Cells were regularly tested and were mycoplasma negative. Cells were seeded at an appropriate density and treated 24 h later with 20 µM MKC8866 (Probechem, St. Pete Beach, USA), 2 µM PF-06424439 (Sigma-Aldrich, PZ0233), 5 µM PF-04620110 (Sigma-Aldrich, PZ0207), 0.5 µg/ml tunicamycin (Tm) (Sigma-Aldrich, T7765), 0.25 µM thapsigargin (Tg) (Sigma-Aldrich, T9033), 0.5 µg/ml brefeldin A (BFA) (Sigma-Aldrich, B6542), 2 µg/ml actinomycin D (Sigma-Aldrich, A9415), or an equal volume of DMSO (Sigma-Aldrich, D2650), unless indicated otherwise.

### Lipid extraction and quantification by LC–MS

Cells were seeded at density 10 × 10^4^, 6 × 10^4^ and 3 × 10^3^ cells/mm^2^. Cells were collected at 24, 48, and 72 h after treatment, washed in ice-cold phosphate-buffered saline (PBS) and snap frozen in liquid nitrogen. Samples were ultrasonicated for 15 min in a 1 ml mixture of methyl-tert-butyl ether (MTBE)/methanol (3:1 v/v) at −15 °C. 2-diheptadecanoyl-sn-glycero-3-phosphocholine was added as an internal standard. Phase separation was obtained by adding 500 µl of water:methanol (3:1 v/v) and centrifugation at 20,000×*g* for 5 min at RT. The upper (organic) phase was moved to a new microfuge tube and evaporated in a vacuum centrifuge. After drying, the samples were dissolved in acetonitrile. The samples were measured with a Waters ACQUITY reversed phase ultra performance liquid chromatography (RP-UPLC) coupled to a Thermo-Fisher Q Exactive Plus mass spectrometer (UPLC–MS). A reversed Phase Bridge Ethyl Hybrid (BEH) C_8_ (100 mm × 2.1 mm × 1.7 μm particles; Waters) column was used for the chromatographic separation. The following mobile phases were used for lipids and lipophilic metabolites separation: 1% of 1 M NH_4_Ac in 0.1% acetic acid (buffer A) and acetonitrile:isopropanol (7:3) containing 1% of 1 M NH_4_Ac in 0.1% acetic acid (buffer B). The separation of lipids was performed with a step gradient from initial 45% buffer A for 1 min, 45% buffer A to 25% buffer A at 4 min, 25% to 11% buffer A at 12 min and 11% to 0% buffer A at 15 min. The column was washed for 4.5 min with 100% buffer B and re-equilibrated for 5 min with 45% buffer A^46^. The mass spectra were acquired in full scan MS positive and negative ionization modes (mass range [100–1500]) for lipid quantitation. Additionally, to extend the annotation, MS/MS spectrum was acquired using data dependent top 3 method. Samples were combined into 10 pools and each was injected once for acquisition in positive and once in negative mode.

For data presented in Fig. 4a–d lipids were extracted from cells with 0.8 ml 1:1 butanol–methanol after media removal^47^. Before extraction, an internal standard (Splash Lipidomix, Avanti Polar Lipids, Alabaster, AL, USA) was added to the extraction buffer and further used as a quality control. Lipids were separated on an Acquity UPLC CSH C18 column (100 × 2.1 mm; 1.7 µm) (Waters corporation, Milford, MA, USA) maintained at 60 °C. The mobile phases consisted of 60:40 ACN:H_2_O with 10 mM ammonium formate, 0.1% formic acid and 5 µM of phosphoric acid (A) and 90:10 IPA:ACN with 10 mM ammonium formate, 0.1% formic acid, and 5 µM phosphoric acid (B). The gradient was as follows: 0–2 min 30% (B); 2–8 min 50% (B); 8–15 min 99% (B), 15–16 min 99% (B), and 16–17 min 30% (B). Sample temperature was maintained at 6 °C in the autosampler and 2 µl of sample were injected.

Analysis of polar and non-polar lipids were conducted using an LC–MS system including an Ultimate 3000 HPLC (Thermo Fisher Scientific, Waltham, MA, USA) coupled to a Q-Exactive Orbitrap mass spectrometer (Thermo Fisher Scientific, Waltham, MA, USA). Q-Exactive Orbitrap MS instrument was operated in both positive and negative polarities, using the following parameters: mass range 240–1200*m*/*z* (positive) and 240–1600 (negative), spray voltage 3.8 kV (ESI+) and 3 kV (ESI−), sheath gas (nitrogen) flow rate 60 units, auxiliary gas (nitrogen) flow rate 25 units, capillary temperature (320 °C), full scan MS1 mass resolving power 70,000. Data dependent fragmentation (dd-MS/MS) parameters for each polarity as follows: TopN: 10, resolution 17,500 units, maximum injection time: 25 ms, automatic gain control target: 5e5 and normalized collision energy of 20 and 25 (arbitrary units) in positive polarity. TopN: 5, resolution 17,500 units, maximum injection time: 80 ms automatic gain control target: 5e5 and normalized collision energy of 20 and 30 (arbitrary units) in negative polarity. The instrument was externally calibrated to <1 ppm using ESI positive and negative calibration solutions (Thermo Fisher Scientific, Waltham, MA, USA).

### Data processing and analysis

Extraction of the LC–MS data presented in Fig. 1, Supplementary Fig. 1 and 2a–c was accomplished with the software REFINER MS^®^ 10.5 (GeneData, http://www.genedata.com). After extraction of the peak list from the chromatograms, the data were processed, aligned and filtered with in-house software. Only those features were selected which were present in at least four out of the five replicates of at least one of the sample groups. The annotation of the extracted features was performed by querying the metaSysX in-house database (619 unique lipid species). The database contains mass-to-charge ratio (*m*/*z*) and retention time information of complex lipids that were previously annotated based on measurements of reference standards or a MS/MS spectra, monoisotopic mass and a retention time shift of consecutive *m*/*z* values. A 5 ppm and 0.06 min deviation from the database mass-to-charge ratio and retention time, respectively, were used as matching criteria. Additionally, lipid annotation was extended with MS/MS fragments spectra of pooled samples using metaSysX-developed R-based algorithm. Pooled samples were measured in dd-MS2 Top 3 mode (Data Dependent tandem mass spectrometry) with the settings: Full Scan MS mode (mass range [100−1500]), NCE 25 (Normalized Collision Energy). Signal intensities were converted into relative abundances by MSTUS normalization^48^, filtered (unannotated, detected in at least half of samples), corrected for batch effect with sva package, log_2_ transformed and row-scaled across samples before performing exploratory multivariate analysis. All data transformations and visualizations were performed in R 4.0.4. Scripts available at: https://github.com/ASAGlab/.

For data presented in Fig. 4a–d peak detection and integration from Raw data were processed using Compound Discoverer 3.0 (Thermo Fisher Scientific, Waltham, MA, USA). Files were also converted to mgf format using MSConvert software and MS2 files were searched against LipidBlast database^49^ (408 unique lipid species) using LipiDex software (Supplementary Data 1 and 2).

### RNA extraction, RT-PCR, and RT-qPCR

Total RNA was isolated using TRI Reagent (Sigma-Aldrich, T9424) according to the manufacturer’s instructions. RNA concentration for each sample was determined with NanoDrop™ 2000 Spectrophotometer (Thermo Scientific) by measuring absorbance at 260 nm. RNA (2 µg) was reverse transcribed using Superscript II (Invitrogen, 18064014). For standard RT-PCR (Supplementary Table 2), products were separated on 1% agarose gels and visualized with DDGIT gel scanner (Li-Core). RT-qPCR reactions were performed using Takyon ROX Master Mix (Eurogentec UFRP5XC0501) and the StepOne Plus platform (Applied Biosystems). Target transcript levels (Supplementary Table 3) were normalized to *MRPL19* and relative abundance was determined using the ΔΔCt method. Transcript-specific TaqMan assays were purchased from Integrated DNA Technologies.

### Cell proliferation

Cells were seeded at density 8 × 10^3^ cells/mm^2^ following indicated treatments for 6 days. Cells were reseeded and treated on day 3. Cell proliferation was monitored by cell count.

### RNA sequencing

MDA-MB-231 cells were seeded at density 2 × 10^4^ cells per mm^2^. RNA was isolated using RNeasy columns (Qiagen, 74104) according to the manufacturer’s protocol. RNA quality was determined by amplifying the 5′ and 3′ ends of the GAPDH transcript upon Oligo dT-primed reverse transcription, and by capillary electrophoresis upon receipt at EMBL. RNA integrity was checked using the Bioanalyzer 2100 system (Agilent Technologies, Santa Clara, CA, USA). A portion of the RNA was taken for pre-analysis. *XBP1* splicing inhibition was confirmed by RT-PCR before the samples were sent to EMBL for analysis. RNA-seq libraries were prepared and sequenced unidirectionally (50 nucleotides read length) with a read depth of 25–50 million reads/library in Illumina sequencing platform at the EMBL Genomics Core Facility in Heidelberg.

### RNA-seq data analysis

Resulting sequenced libraries were assessed in a quality control step with FastQC (available from https://www.bioinformatics.babraham.ac.uk/projects/fastqc/), followed by adapter sequences trimming with TrimGalore (http://www.bioinformatics.babraham.ac.uk/projects/trim_galore). Reads were aligned with reference genome using STAR. Reads/counts per gene were summarized with FeatureCounts^50^. The computational-heavy steps were performed using the cloud-computing infrastructure of SevenBridges. Genes with low counts were filtered out with limma^51^. Counts were normalized between samples by the method of trimmed mean of M-values (TMM), dataset corrected for batch effect with sva package^52^. Statistical inference of differentially expressed genes was carried out with edgeR^53^ by performing pairwise comparison between DMSO and MKC8866 treated samples at both time points. Genes with a *p* value below 0.05 and a minimum log_2-_fold change of the mean of 0.25 were considered as significantly changing. Ggplot2, pheatmap packages at R/Bioconductor were used for graphical representation. Functional analysis of the significantly changing genes was performed with BioInfominer, utilizing Gene Ontology Biological Processes annotation corpus^24^. Overrepresented GO terms were summarized into broader biological groups based on a semantic similarity with cateGOrizer^25^. All data transformations and visualizations were performed in R 4.0.4. Scripts available at: https://github.com/ASAGlab/.

### Western blotting

Cells were washed once in ice-cold 1× PBS and lysed in radioimmunoprecipitation assay (RIPA) buffer (0.1% SDS, 1% NP-40, 0.5% sodium deoxycholate, 50 mM Tris–HCl pH 8.8, 150 mM NaCl) after indicated treatments and cell lysates were boiled at 95 **°**C for 5 min. Protein samples were separated on an SDS polyacrylamide gel, transferred onto nitrocellulose membrane (Amersham Protran 0.2 10600001) and blocked with 5% milk in PBS-0.1% Tween. For detection of protein expression the following antibodies were used: IRE1α (Cell Signaling Technology, 3294, 1:1,000), XBP1s (Biolegend, 647502, 1:1,000), FLAG (Sigma-Aldrich, F1804; 1:1,000) and Actin (Sigma-Aldrich, A-5060, 1:5000). Anti-rabbit (111-035-003) and anti-mouse (115-035-003) HRP-conjugated secondary antibodies were purchased from Jackson Immunoresearch and the signal was visualized using Western Blotting Luminol Reagent (SantaCruz, sc-2048).

### Transient knockdown

For knockdown, MDA-MB-231 cells were transfected with 25 nM of Dharmacon On-Target SMARTpool Plus siRNA targeting IRE1 (Dharmacon, L-004951-02), or non-targeting control (nc) siRNA (Dharmacon, D-001810-01-20) using Dharmafect 4 (Dharmacon T-2004-02) according to the manufacturer’s instructions.

### *XBP1* knockout in MDA-MB-231 cells

MDA-MB-231 were transfected with CRISPR scramble guide RNA (gRNA) plasmid (OriGene, #GE100003) or gRNA targeting XBP1 sequence CCCGTCGGCCGGGTTCGGCG ((OriGene, #KN201959G2) in combination with the donor DNA (OriGene, #KN201959-D). TurboFectin transfection reagent (OriGene #TF81001) was used according to the manufacturer’s instructions with a 1:2 ratio of DNA to transfection reagent. Pooled cells were selected using 500 ng/ml puromycin (Sigma, P8833). Single clones were isolated and positive clones were confirmed by Western blot.

### In vitro *DGAT2* mRNA cleavage assay

pcDNA3.1 plasmids expressing full length *DGAT2* (CCDS31642.1), a c.260G>A mutated version of *DGAT2* and unspliced *XBP1* were purchased from GenScript. Plasmids were linearized by digestion with *SmaI* restriction enzyme followed by removal of salts and enzymes with a column-based Nucleospin PCR cleanup system (Macherey-Nagel, 740609.50) according to the manufacturer’s protocol. The linearized plasmids served as templates for in vitro transcription using T7 RiboMAX Express Large Scale RNA Production System (Promega, P1320). RNA was purified with Nucleospin PCR cleanup system. RNA (1 µg) was incubated with recombinant IRE1α-N-His-GST fragment containing the cytosolic kinase and endoribonuclease domains (aa 465-977) (Creative Biomart, ERN1-1124H) in the presence or absence of 20 μM MKC8866 for 1 h at 37 °C in a buffer containing 40 mM HEPES pH 7.5, 2 mM MgOAc_2_, 100 mM KOAc, 1 mM DTT. Products of the digestion were separated in a 1.5% agarose gel made in 1× MOPS (Fisher Scientific, BP2900-1) and visualized with DDGIT gel scanner (LI-COR).

### Stable *DGAT2* overexpression

pGenLenti plasmids expressing full length *DGAT2* (CCDS31642.1) and a c.260G>A mutated version of *DGAT2* were purchased from GenScript. Lentivirus was generated by co-transfecting the above plasmids with second-generation lentivirus-packaging system (Addgene, pMD2.G 12259; psPAX2, 12260; pRSV-Rev, 12253) using PEI Prime transfection reagent (Sigma-Aldrich, 919012) into HEK293T cells. Virus-containing supernatant was harvested and filtered through 0.22 μm filter (Sarstedt Filtropur 83.1826.001). MDA-MB-231 cells were transduced with this media and selected in 400 ng/ml of puromycin (Sigma-Aldrich, P8833).

### Nile red staining

Nile red is a lipophilic fluorescent dye that accumulates in hydrophobic environments in cells thus enabling visualization of lipid droplets (LDs). Cells were seeded at 8 × 10^3^ cells per mm^2^ and treated with 20 μM MKC8866 in the presence or absence of 2 µM PF-06424439 and 5 µM PF-04620110 for up to 6 days. For the 6 days incubation, MDA-MB-231 cells were reseeded and treated after 3 days. HCC1806 and MCF10A were treated every second day and reseeded after 4 days. At the required time point, cells were fixed with 4% paraformaldehyde for 15 min, washed twice with PBS and stained with 2 µg/ml Nile red in PBS (Sigma-Aldrich, 19123) for 1 h in the dark. Stained cells were washed three times with PBS. Phase contrast and fluorescence images were collected at a single time-point by IncuCyte (Sartorius). Cell-by-cell analysis and cell classification were performed with Incucyte 2019B Rev2 image analysis software.

### Mito stress test

Cells were seeded at a density of 8 × 10^3^ cells/mm^2^ and incubated with drugs for up to 6 days. MDA-MB-231 cells were reseeded and treated at day 3 and 5. At day 5, MDA-MB-231 cells were reseeded into XFp Cell Culture Miniplates (Agilent, 103025-100) at density 8 × 10^3^ cells per well. HCC1806 and MCF10A were treated every second day and reseeded at day 4. XF Cell Mito stress test (Agilent, 103010-100) was performed at day 6 according the manufacturer’s protocol. The oxygen consumption rate (OCR) was measured using a Seahorse XFp analyzer (Agilent) under basal conditions and after injection of 1 µM oligomycin, 0.5 μM carbonyl-cyanide-4-(trifluoromethoxy)phenylhydrazone (FCCP), and 0.5 µM rotenone/antimycin A. Mitochondrial respiration parameters were calculated using the Seahorse Bioscience Wave software. The calculated parameters included: basal respiration (last OCR measurement before first oligomycin injection minus minimum OCR measurement post rotenone/antimycin A injection), ATP production (last OCR measurement before first oligomycin injection minus minimum OCR measurement after oligomycin injection), maximal respiratory capacity (post FCCP respiration minus OCR after rotenone/antimycin A injection), spare respiratory capacity (SCR, maximal respiration/basal respiration × 100), non-mitochondrial oxygen consumption (minimum rate measurement after rotenone/antimycin A injection) and proton leak (minimum rate measurement after oligomycin injection minus non-mitochondrial respiration).

### Assessment of cell death kinetics using Sytox Green

Cells were incubated with 20 μM MKC8866 in the presence or absence of 2 μM PF-06424439 for up to 6 days. MDA-MB-231 cells were reseeded and treated at day 3 and 5. HCC1806 and MCF10A cells were treated every second day and reseeded at day 4. For Sytox Green uptake assay cells were reseeded into 96-well plates at density 8 × 10^3^ cells/mm^2^ and treated with 20 μM MKC8866 in the presence or absence of 2 µM PF-06424439. After 24 h, the culture medium was replaced either with complete DMEM medium or Hank’s balanced salt solution (Sigma, H9394) containing 0.5 μM Sytox Green (Life Technologies, S7020). Cells were imaged at 2 h intervals for 72 h using the IncuCyte S3 live cell analysis system (Sartorius). Four regions per well were captured at ×10 magnification using bright field and green fluorescence channel (excitation 504 nm and emission 523 nm). The percentage of Sytox Green-positive cells was quantified using IncuCyte v2019B software.

### Statistical analysis

Assumptions concerning the data (normal distribution and similar variation between experimental groups) were examined for appropriateness before statistical tests were conducted. Statistical analysis was carried out using one-way or two-way ANOVA with Bonferroni multiple comparisons post hoc test, unpaired two-tailed Student’s *t*-test and one-sample *t*-test (comparison value of zero) as indicated. Values with *p* < 0.05 are considered statistically significant.

### Reporting summary

Further information on research design is available in the Nature Research Reporting Summary linked to this article.

## Supplementary information

Supplementary Information

Description of Additional Supplementary Files

Supplementary Data 1

Supplementary Data 2

Reporting Summary

## Data Availability

All data supporting the findings of this study are available at: https://github.com/ASAGlab. https://github.com/ASAGlab/Regulated-IRE1-dependent-decay-RIDD-mediated-reprograming-of-lipid-metabolism-in-cancer or from the corresponding authors upon request. The RNA sequencing data have been deposited and are available in the Gene Expression Omnibus (GEO) database under accession number GSE176454. Metabolomics data are included in this article as excel tables in Supplementary Data. Source data are provided with this paper.

## Source data

Source Data

## Code availability

The code used for the analysis is available at: https://github.com/ASAGlab/Regulated-IRE1-dependent-decay-RIDD-mediated-reprograming-of-lipid-metabolism-in-cancer^54^. All computational analyses were performed in R (version 4.0.4).
