# Supplementary material for: Regulated IRE1α-dependent decay (RIDD)-mediated reprograming of lipid metabolism in cancer
Source: Nat Commun. 2022 May 6;13:2493. doi: 10.1038/s41467-022-30159-0 (PMC9076827; doi:10.1038/s41467-022-30159-0)
Supplement: Supplementary file 1 — Supplementary Information [file 41467_2022_30159_MOESM1_ESM.pdf]

Supplementary Information

Regulated IRE1α-dependent decay (RIDD)-mediated reprograming of lipid metabolism in cancer

Almanza and Mnich *et al*

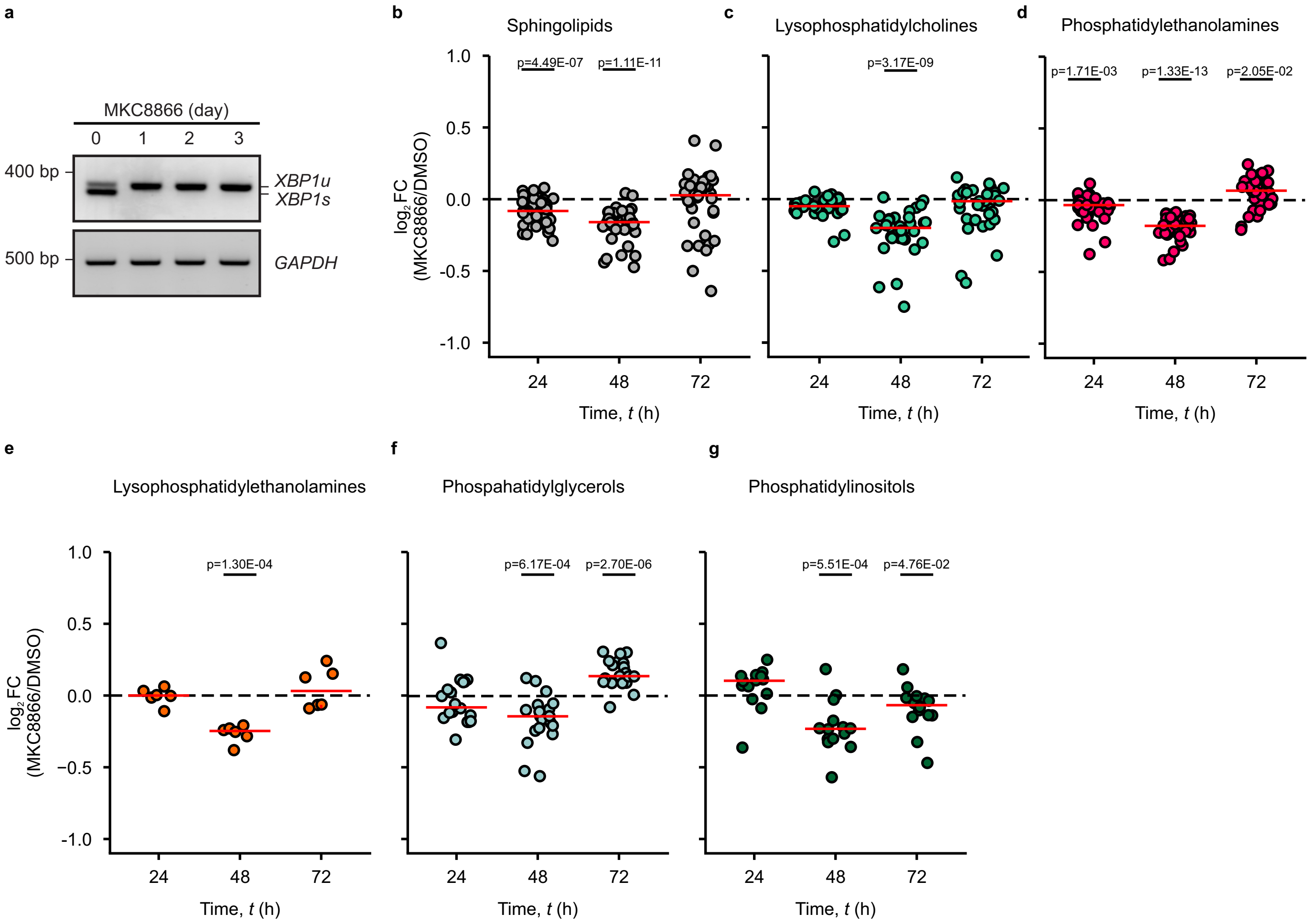

Supplementary Fig. 1 IRE1α inhibition alters lipid metabolism in MDA-MB-231 cells. a-g MDA-MB-231 cells were treated with 20 μM MKC8866 or vehicle (DMSO) for 24, 48 and 72 h. **a** Representative image of RT-PCR analysis of *XBP1u, XBP1s* and *GAPDH* mRNA expression (n=3 biologically independent experiments). Sample processing controls were run on different gels. **b-g** Scatter plots with mean of $\log_2$ fold changes ($\log_2$ FC) (MKC8866/DMSO) in levels of **b** sphingolipid (n=40), **c** lysophosphatidylcholine (n=34), **d** phosphatidylethanolamine (n=32), **e** lysophosphatidylethanolamine (n=6), **f** phosphatidylglycerol (n=19), **g** phosphatidylinositol (n=14) species over the indicated time points (n=5 biologically independent experiments). Statistical comparisons were performed with unpaired one-sample t-test to determine whether the population mean is different from 0. P values indicated on figures. Source data are provided as a Source Data file.

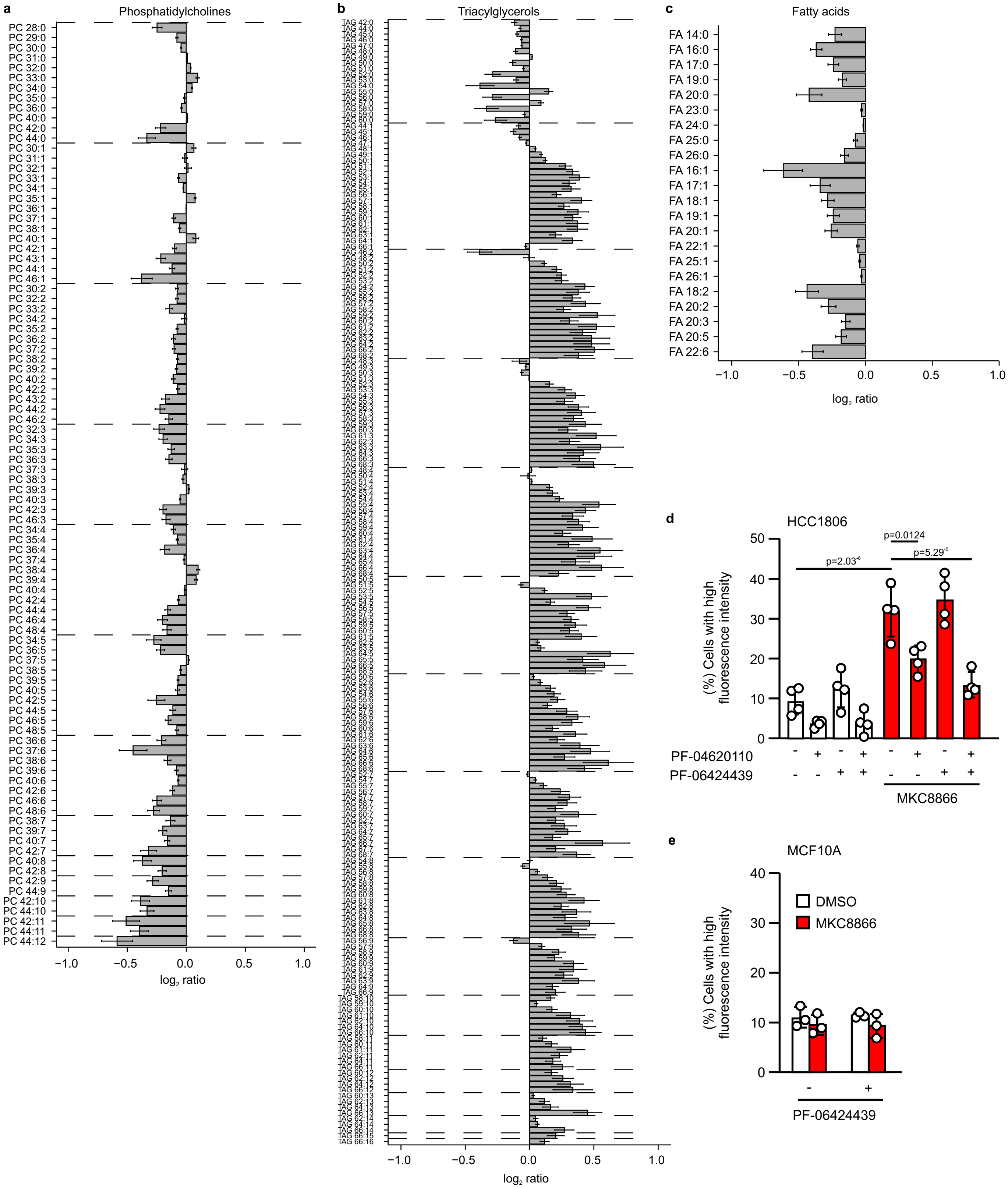

Supplementary Fig. 2 MKC8866 treatment causes a shift towards the usage of elongated unsaturated lipids. a-c MDA-MB-231 cells were treated with 20 μM MKC8866 or vehicle (DMSO) for 24, 48 and 72 h, after which lipids were extracted and their levels were analysed by liquid chromatography tandem mass spectrometry (LC-MS) (n=5 biologically independent experiments). Bar charts showing $\log_2$ FC (mean of MKC8866 group /mean of DMSO group) ± s.e.m. estimated by propagation of error for each individual lipid from the **a** phosphatidylcholine, **b** triacylglycerol and **c** fatty acid lipid groups. For duplicated entries annotated to the same lipid species the mean value was taken. **d** HCC1806 cells (n=4 biologically independent experiments) and **e** MCF10A cells (n=3 biologically independent experiments) were treated with 20 μM MKC8866 for 6 days in the presence or absence of 5 μM PF-04620110 or 2 μM PF-06424439 as indicated and the percentage of cells with high LD content is shown. Indicated p values based on one-way ANOVA with Bonferroni's multiple comparisons *post hoc* tests. Data are presented as mean values ± s.d. Source data are provided as a Source Data file.

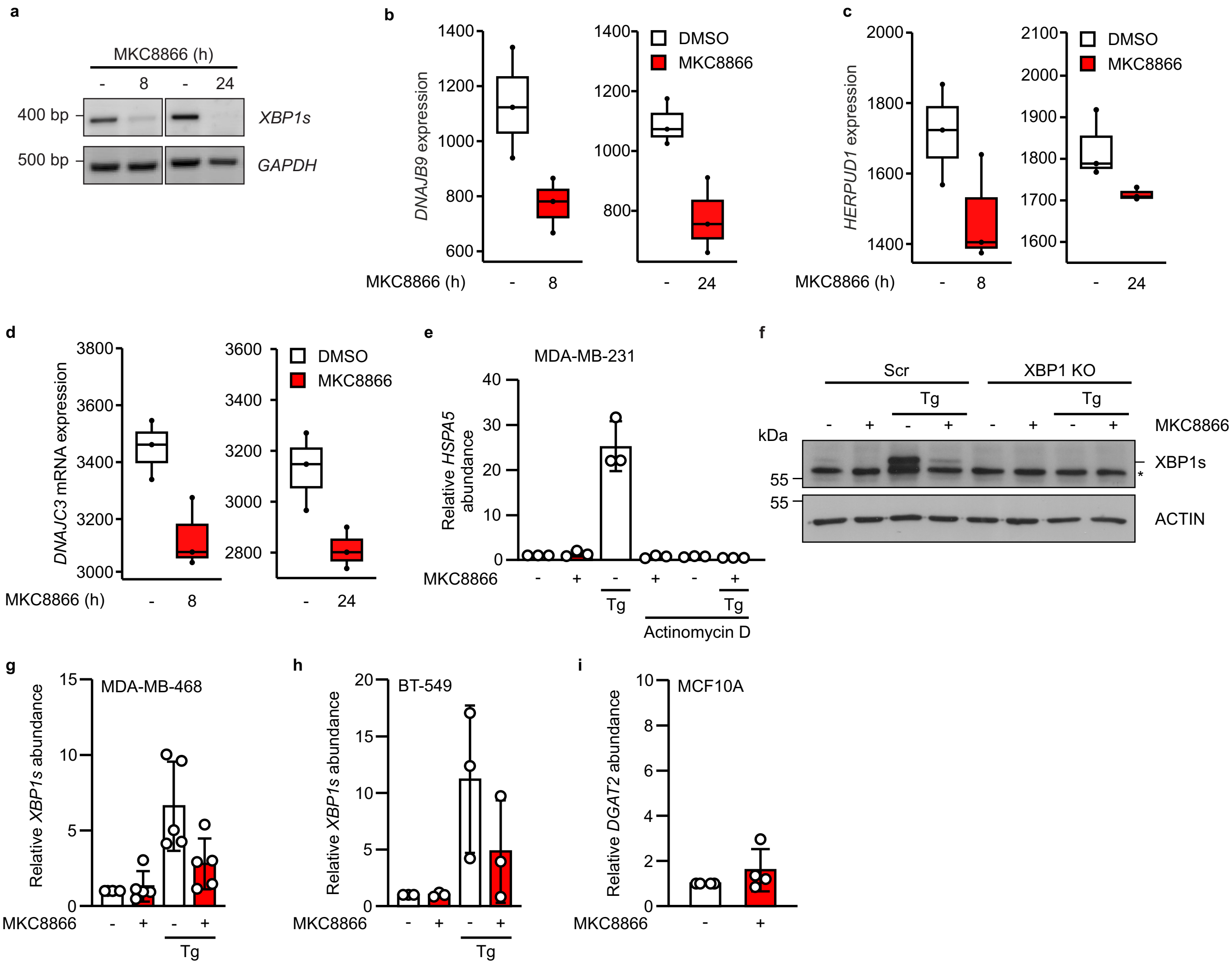

Supplementary Fig. 3 Regulation of IRE1α activity alters gene expression in TNBC cells. a-d MDA-MB-231 cells were treated with 20 μM MKC8866 for 8 h and 24 h, after which RNA was extracted and RNA sequencing analysis performed (n=3 biologically independent experiments). **a** RT-PCR analysis of *XBP1s* and *GAPDH* mRNA expression. Sample processing controls were run on different gels. **b-d** Expression levels of known XBP1s-regulated genes **b** *DNAJB9,* **c** *HERPUD1* and **d** *DNAJC3*. Y axis indicates normalized and batch effect-adjusted RNAseq counts. Boxplots displaying median (middle line), upper and lower quartiles (box), while whiskers stretch to maxima and minima. **e** MDA-MB-231 cells were pre-treated with or without actinomycin D (2 μg/ml) for 2 h followed by incubation of cells with Tg (0.25 μM) and MKC8866 (20 μM) for 8 h (n=3 biologically independent experiments). RT-qPCR analysis of *HSPA5* expression relative to *ACTB* and normalised to control. **f** XBP1 KO MDA-MB-231 cells and their control counterparts (Scr) were incubated with DMSO or 20 μM MKC8866 in the presence or absence of 1 μM Tg for 24 h (n=3 biologically independent experiments). Immunoblotting of XBP1s and ACTIN. * indicates a non-specific band. **g** MDA-MB-468 (n=5 biologically independent experiments), **h** BT-549 (n=3 biologically independent experiments) and **i** MCF10A cells (n=4 biologically independent experiments) were treated with 20 μM MKC8866 and 1 μM Tg for 24 h. RT-qPCR analysis of **g-h** *XBP1s* and **i** *DGAT2* expression relative to *MRPL19* (**g** and **h**) or *ACTB* (**i**) and normalised to control. Data are presented as mean values ± s.d. Source data are provided as a Source Data file.

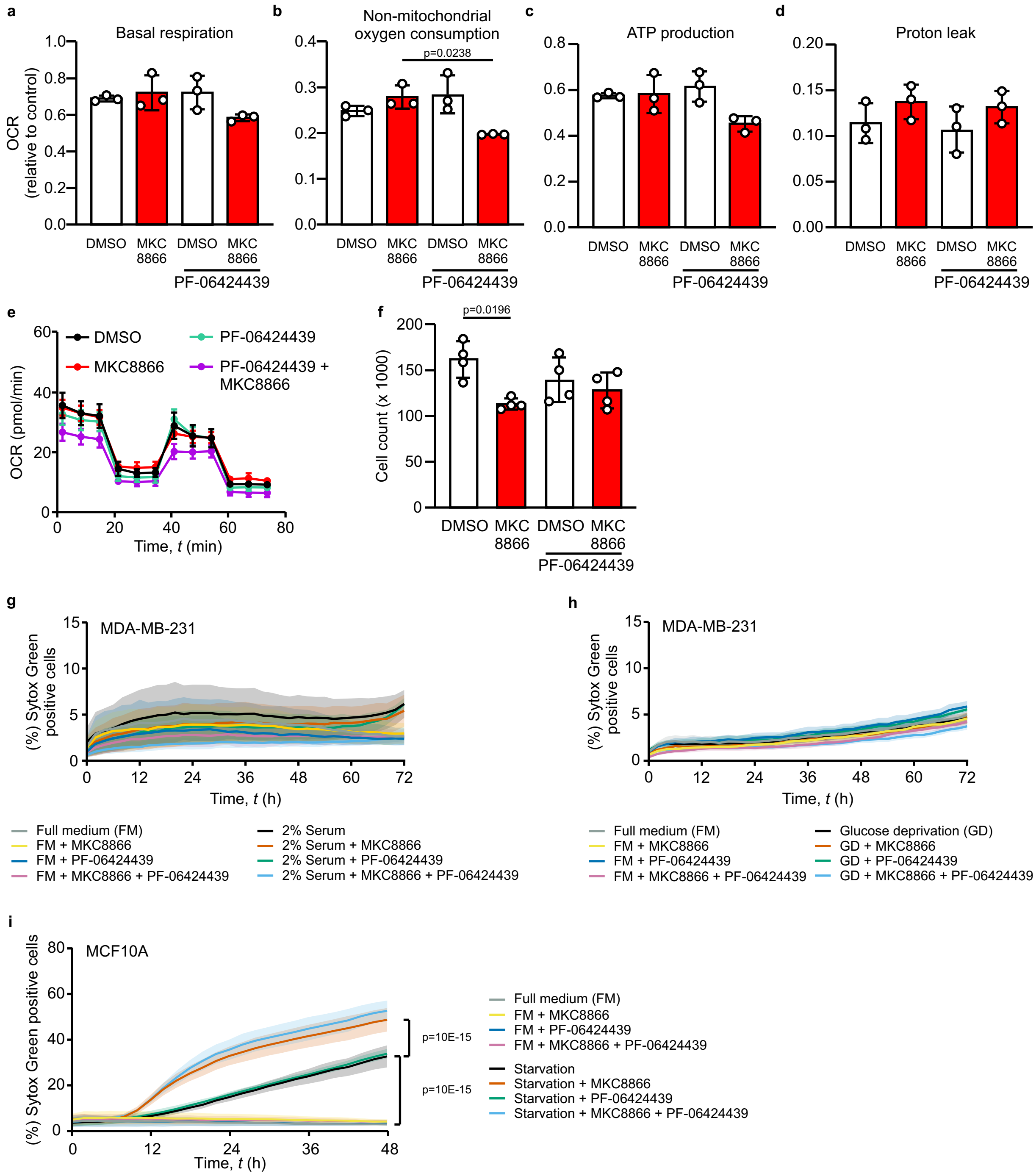

Supplementary Fig. 4 a-e Seahorse extracellular flux analysis of **a-d** MDA-MB-231 (n=3 biologically independent experiments) and **e** HCC1806 cells (n=3 biologically independent experiments) treated with vehicle or 20 μM MKC8866 in the presence or absence of 2 μM PF-06424439 for 6 days. **a-d** Normalized data plotted to demonstrate **a** basal respiration, **b** non-mitochondrial oxygen consumption rate, **c** ATP production, **d** proton leak. **e** Data plotted to demonstrate oxygen consumption rate. **f** MDA-MB-231 cells were treated with vehicle or 20 μM MKC8866 in the presence or absence of 2 μM PF-06424439 for 6 days (n=4 biologically independent experiments). Cell proliferation monitored by cell count. Plotted p values based on one-way ANOVA with Bonferroni's multiple comparisions *post hoc* tests. **g-h** MDA-MB-231 cells or **i** MCF10A cells were treated with vehicle or 20 μM MKC8866 in the presence or absence of 2 μM PF-06424439. After 6 days, culture medium was replaced with a complete medium or **g** medium containing 2% FBS (n=4 biologically independent experiments), **h** medium without glucose (n=3 biologically independent experiments) or **i** Hanks' balanced salt solution (n=3 biologically independent experiments). Kinetics of cell death expressed as the percentage of cells that were Sytox Green positive. Plotted p values based on two-way ANOVA with Bonferroni's multiple comparisions *post hoc* tests. Values with p<0.05 are considered statistically significant. Data are presented as mean values **a-d, f** ± s.d. or **e, g-i** ± s.e.m. Source data are provided as a Source Data file.

8 hours

Term id	Term Definition	Enrichment	Corrected pvalue
GO:0003241	growth involved in heart morphogenesis	3/5	4.00E-04
GO:0045824	negative regulation of innate immune response	8/57	1.60E-03
GO:0070245	positive regulation of thymocyte apoptotic process	3/6	1.70E-03
GO:0010867	positive regulation of triglyceride biosynthetic process	4/14	1.70E-03
GO:0010875	positive regulation of cholesterol efflux	4/16	1.70E-03
GO:0010874	regulation of cholesterol efflux	6/39	3.00E-03
GO:0000122	negative regulation of transcription by RNA polymerase II	47/831	3.10E-03
GO:0045995	regulation of embryonic development	12/132	3.20E-03
GO:0051491	positive regulation of filopodium assembly	5/30	5.00E-03
GO:0008593	regulation of Notch signaling pathway	10/101	5.30E-03
GO:0003211	cardiac ventricle formation	3/10	5.70E-03
GO:0045892	negative regulation of transcription, DNA-templated	62/1188	7.00E-03
GO:0051128	regulation of cellular component organization	116/2465	7.10E-03
GO:1902679	negative regulation of RNA biosynthetic process	64/1243	7.20E-03
GO:1903507	negative regulation of nucleic acid-templated transcription	64/1241	7.30E-03
GO:0032368	regulation of lipid transport	11/127	8.10E-03
GO:0090208	positive regulation of triglyceride metabolic process	4/21	9.90E-03
GO:0030010	establishment of cell polarity	10/111	1.00E-02
GO:0051253	negative regulation of RNA metabolic process	67/1329	1.02E-02
GO:0070830	bicellular tight junction assembly	5/34	1.06E-02
GO:0042059	negative regulation of epidermal growth factor receptor signaling pathway	6/48	1.16E-02
GO:0060174	limb bud formation	3/12	1.18E-02
GO:0060547	negative regulation of necrotic cell death	4/24	1.18E-02
GO:0051004	regulation of lipoprotein lipase activity	4/23	1.26E-02
GO:0006801	superoxide metabolic process	5/37	1.38E-02
GO:0001568	blood vessel development	29/496	1.38E-02
GO:0050777	negative regulation of immune response	12/150	1.39E-02
GO:0120034	positive regulation of plasma membrane bounded cell projection assembly	9/99	1.43E-02
GO:1901879	regulation of protein depolymerization	8/83	1.48E-02
GO:0045747	positive regulation of Notch signaling pathway	6/52	1.55E-02
GO:0010817	regulation of hormone levels	30/528	1.59E-02
GO:0042147	retrograde transport, endosome to Golgi	8/84	1.64E-02
GO:0001960	negative regulation of cytokine-mediated signaling pathway	7/67	1.68E-02
GO:1901185	negative regulation of ERBB signaling pathway	6/53	1.73E-02
GO:0007005	mitochondrion organization	26/442	1.78E-02
GO:0016197	endosomal transport	15/216	1.91E-02
GO:0072655	establishment of protein localization to mitochondrion	7/72	1.98E-02
GO:0048145	regulation of fibroblast proliferation	8/88	1.99E-02
GO:0060761	negative regulation of response to cytokine stimulus	7/71	2.02E-02
GO:0045746	negative regulation of Notch signaling pathway	5/40	2.18E-02
GO:1905952	regulation of lipid localization	12/158	2.21E-02
GO:0045934	negative regulation of nucleobase-containing compound metabolic process	69/1427	2.21E-02
GO:0001944	vasculature development	29/517	2.29E-02
GO:0050796	regulation of insulin secretion	13/181	2.34E-02
GO:0044058	regulation of digestive system process	5/42	2.41E-02
GO:0007219	Notch signaling pathway	10/128	2.44E-02
GO:0001649	osteoblast differentiation	10/126	2.45E-02
GO:0001959	regulation of cytokine-mediated signaling pathway	12/167	2.54E-02
GO:0051235	maintenance of location	12/163	2.56E-02
GO:0090181	regulation of cholesterol metabolic process	6/59	2.67E-02
GO:0034330	cell junction organization	14/208	2.72E-02
GO:0050654	chondroitin sulfate proteoglycan metabolic process	5/44	2.85E-02
GO:0072358	cardiovascular system development	29/527	2.94E-02
GO:0006915	apoptotic process	46/920	2.97E-02
GO:0051489	regulation of filopodium assembly	5/45	3.00E-02
GO:0042269	regulation of natural killer cell mediated cytotoxicity	5/46	3.01E-02
GO:0043433	negative regulation of DNA-binding transcription factor activity	12/171	3.04E-02
GO:0007163	establishment or maintenance of cell polarity	13/189	3.08E-02
GO:0042632	cholesterol homeostasis	8/96	3.08E-02
GO:0002716	negative regulation of natural killer cell mediated immunity	3/17	3.12E-02
GO:0033043	regulation of organelle organization	61/1275	3.27E-02
GO:1904035	regulation of epithelial cell apoptotic process	8/95	3.30E-02
GO:0045446	endothelial cell differentiation	7/80	3.39E-02
GO:0045216	cell-cell junction organization	10/133	3.45E-02
GO:1901880	negative regulation of protein depolymerization	6/62	3.58E-02
GO:0006505	GPI anchor metabolic process	4/32	3.59E-02
GO:0120032	regulation of plasma membrane bounded cell projection assembly	12/177	3.59E-02
GO:0031343	positive regulation of cell killing	6/65	3.60E-02
GO:0090276	regulation of peptide hormone secretion	14/214	3.62E-02
GO:0006638	neutral lipid metabolic process	8/98	3.64E-02
GO:0043542	endothelial cell migration	6/64	3.65E-02
GO:0060560	developmental growth involved in morphogenesis	9/118	3.80E-02
GO:0002715	regulation of natural killer cell mediated immunity	5/48	3.95E-02
GO:0009612	response to mechanical stimulus	14/218	4.01E-02
GO:0060491	regulation of cell projection assembly	12/179	4.05E-02
GO:0031334	positive regulation of protein complex assembly	15/241	4.05E-02
GO:0060759	regulation of response to cytokine stimulus	12/178	4.07E-02
GO:0019218	regulation of steroid metabolic process	9/121	4.13E-02
GO:0035265	organ growth	8/103	4.20E-02
GO:0045582	positive regulation of T cell differentiation	7/83	4.21E-02
GO:0046503	glycerolipid catabolic process	5/51	4.23E-02
GO:0051493	regulation of cytoskeleton organization	28/535	4.33E-02
GO:0001667	ameboidal-type cell migration	11/167	4.52E-02
GO:0090100	positive regulation of transmembrane receptor protein serine/threonine kinase signaling pathway	8/104	4.58E-02
GO:0015914	phospholipid transport	7/87	4.63E-02
GO:0070125	mitochondrial translational elongation	7/88	4.64E-02
GO:0043062	extracellular structure organization	21/380	4.76E-02
GO:0009749	response to glucose	10/146	4.79E-02
GO:0009967	positive regulation of signal transduction	76/1678	4.80E-02
GO:0043242	negative regulation of protein complex disassembly	6/69	4.81E-02
GO:0034329	cell junction assembly	11/163	4.86E-02
GO:0031146	SCF-dependent proteasomal ubiquitin-dependent protein catabolic process	7/90	4.94E-02
GO:1903509	liposaccharide metabolic process	8/108	4.96E-02

24 hours

Term id	Term Definition	Enrichment	Corrected pvalue
GO:1902512	positive regulation of apoptotic DNA fragmentation	2/4	2.00E-04
GO:0015813	L-glutamate transmembrane transport	3/19	1.70E-03
GO:1903626	positive regulation of DNA catabolic process	2/5	2.70E-03
GO:0009220	pyrimidine ribonucleotide biosynthetic process	3/22	2.70E-03
GO:0002476	antigen processing and presentation of endogenous peptide antigen via MHC class Ib	2/7	4.20E-03
GO:0009218	pyrimidine ribonucleotide metabolic process	3/24	4.50E-03
GO:0030148	sphingolipid biosynthetic process	5/90	4.90E-03
GO:0006220	pyrimidine nucleotide metabolic process	4/62	5.60E-03
GO:0019933	cAMP-mediated signaling	6/147	6.40E-03
GO:0060536	cartilage morphogenesis	3/29	6.60E-03
GO:0034308	primary alcohol metabolic process	4/72	7.10E-03
GO:0034381	plasma lipoprotein particle clearance	3/40	7.30E-03
GO:0046520	sphingoid biosynthetic process	2/12	7.80E-03
GO:0019935	cyclic-nucleotide-mediated signaling	6/170	9.60E-03
GO:0007568	aging	8/282	1.03E-02
GO:0003333	amino acid transmembrane transport	4/77	1.10E-02
GO:0006805	xenobiotic metabolic process	5/122	1.14E-02
GO:1902475	L-alpha-amino acid transmembrane transport	16132	1.22E-02
GO:0006766	vitamin metabolic process	5/123	1.24E-02
GO:0071466	cellular response to xenobiotic stimulus	6/181	1.52E-02
GO:0017144	drug metabolic process	11/487	1.52E-02
GO:0015800	acidic amino acid transport	3/47	1.56E-02
GO:0046467	membrane lipid biosynthetic process	5/130	1.69E-02
GO:0002716	negative regulation of natural killer cell mediated immunity	2/17	1.79E-02
GO:0070542	response to fatty acid	4/91	1.85E-02
GO:0002715	regulation of natural killer cell mediated immunity	3/48	1.93E-02
GO:0006643	membrane lipid metabolic process	6/199	1.99E-02
GO:0042445	hormone metabolic process	6/196	2.03E-02
GO:0001912	positive regulation of leukocyte mediated cytotoxicity	3/55	2.19E-02
GO:0006979	response to oxidative stress	9/389	2.22E-02
GO:0006665	sphingolipid metabolic process	5/151	2.25E-02
GO:0060548	negative regulation of cell death	18/1037	2.39E-02
GO:1903076	regulation of protein localization to plasma membrane	4/99	2.40E-02
GO:0015807	L-amino acid transport	3/57	2.52E-02
GO:0072527	pyrimidine-containing compound metabolic process	4/100	2.60E-02
GO:0030154	cell differentiation	50/3683	2.65E-02
GO:1903078	positive regulation of protein localization to plasma membrane	3/58	2.73E-02
GO:0019932	second-messenger-mediated signaling	8/335	2.78E-02
GO:0046939	nucleotide phosphorylation	3/61	2.89E-02
GO:0007584	response to nutrient	6/221	2.90E-02
GO:0048869	cellular developmental process	50/3776	2.91E-02
GO:0001676	long-chain fatty acid metabolic process	4/108	3.01E-02
GO:0019751	polyol metabolic process	4/110	3.13E-02
GO:0048638	regulation of developmental growth	8/351	3.16E-02
GO:0009410	response to xenobiotic stimulus	7/295	3.20E-02
GO:0010941	regulation of cell death	26/1749	3.33E-02
GO:0007127	meiosis I	4/109	3.38E-02
GO:0044331	cell-cell adhesion mediated by cadherin	2/27	3.43E-02
GO:1904375	regulation of protein localization to cell periphery	4/117	3.50E-02
GO:0006865	amino acid transport	4/119	3.62E-02
GO:0006836	neurotransmitter transport	5/178	3.67E-02
GO:0009636	response to toxic substance	10/510	3.76E-02
GO:0034599	cellular response to oxidative stress	6/239	3.87E-02
GO:0010469	regulation of signaling receptor activity	11/600	3.89E-02
GO:1901657	glycosyl compound metabolic process	4/130	4.04E-02
GO:0007189	adenylate cyclase-activating G protein-coupled receptor signaling pathway	4/128	4.14E-02
GO:1901615	organic hydroxy compound metabolic process	9/444	4.21E-02
GO:0006066	alcohol metabolic process	7/304	4.23E-02
GO:0046677	response to antibiotic	7/317	4.23E-02
GO:1901135	carbohydrate derivative metabolic process	16/986	4.26E-02
GO:0001910	regulation of leukocyte mediated cytotoxicity	3/77	4.39E-02
GO:0098656	anion transmembrane transport	6/252	4.46E-02
GO:0034332	adherens junction organization	3/83	4.57E-02
GO:0070588	calcium ion transmembrane transport	5/199	4.65E-02
GO:0090150	establishment of protein localization to membrane	6/262	4.67E-02
GO:0008219	cell death	17/1085	4.72E-02
GO:0071248	cellular response to metal ion	5/192	4.74E-02

Supplementary Table 1 Functional analysis of the significantly changing genes followed by MKC8866 treatment for 8 and 24 h.

Transcript		Sequence (5’ – 3’)
Total XBP1	FWD<br>REV	GGAACAGCAAGTGGTAGA<br>CTGGAGGGGTGACAACTG
XBP1s	FWD<br>REV	TCTGCTGAGTCCGCAGCAGG<br>CTCTAAGACTAGAGGCTTGG
XBP1u	FWD<br>REV	CAGACTACGTGCGCCTCTGC<br>CTTCTGGGTAGACTTCTGGG
GAPDH	FWD<br>REV	ACCACAGTCCATGCCATC<br>TCCACCACCCTGTTGCTG

Supplementary Table 2 PCR primer sequences

**Transcript**		**Sequence (5'– 3')**
MRPL19	FWD	CTTAGGAATGTTATCGAAGGACAAG
	REV	GCTATATTCAGGAAGGGCATCT
	PROBE	CTCGGGTCCAGGAGAGATTCAGGTG
ACTB	FWD	ACAGAGCCTCGCCTTTG
	REV	CCTTGCACATGCCGGAG
	PROBE	TCATCCATGGTGAGCTGGCGG
DGAT2	FWD	GGCTGGTGTTTGACTGGAA
	REV	TCAGCAGGTTGTGTGTCTTC
	PROBE	CCAAGAAAGGTGGCAGGAGGTCA
HSPA5	FWD	CCAGTCAGATCAAATGTACCCA
	REV	GTGCCTACCAAGAAGTCTCAG
	PROBE	TTGTCTTTTGTCAGGGGTCTTTCACCT

Supplementary Table 3 RT-qPCR primer sequences
