# Supplementary material for: Regulated IRE1α-dependent decay (RIDD)-mediated reprograming of lipid metabolism in cancer
Source: Nat Commun. 2022 May 6;13:2493. doi: 10.1038/s41467-022-30159-0 (PMC9076827; doi:10.1038/s41467-022-30159-0)
Supplement: Supplementary file 2 — Description of Additional Supplementary Files [file 41467_2022_30159_MOESM2_ESM.pdf]

Description of Additional Supplementary Files

File Name: Supplementary Data 1
Description: Tables containing sample information and measurements for the mass spectrometry based metabolomic profiling experiment underlying figure 1

File Name: Supplementary Data 2
Description: Tables containing sample information and measurements for the mass spectrometry based metabolomic profiling experiment underlying figure 4
