# Supplementary material for: Regulated IRE1α-dependent decay (RIDD)-mediated reprograming of lipid metabolism in cancer
Source: Nat Commun. 2022 May 6;13:2493. doi: 10.1038/s41467-022-30159-0 (PMC9076827; doi:10.1038/s41467-022-30159-0)
Supplement: Supplementary file 5 — Reporting Summary [file 41467_2022_30159_MOESM5_ESM.pdf]

Corresponding author(s): Afshin Samali
Last updated by author(s): 15-03-2022

Reporting Summary

Nature Portfolio wishes to improve the reproducibility of the work that we publish. This form provides structure for consistency and transparency in reporting. For further information on Nature Portfolio policies, see our Editorial Policies and the Editorial Policy Checklist.

Statistics

For all statistical analyses, confirm that the following items are present in the figure legend, table legend, main text, or Methods section.

n/a	Confirmed	
☐	☒	The exact sample size (*n*) for each experimental group/condition, given as a discrete number and unit of measurement
☐	☒	A statement on whether measurements were taken from distinct samples or whether the same sample was measured repeatedly
☐	☒	The statistical test(s) used AND whether they are one- or two-sided<br>*Only common tests should be described solely by name; describe more complex techniques in the Methods section.*
☐	☒	A description of all covariates tested
☐	☒	A description of any assumptions or corrections, such as tests of normality and adjustment for multiple comparisons
☐	☒	A full description of the statistical parameters including central tendency (e.g. means) or other basic estimates (e.g. regression coefficient) AND variation (e.g. standard deviation) or associated estimates of uncertainty (e.g. confidence intervals)
☒	☐	For null hypothesis testing, the test statistic (e.g. *F*, *t*, *r*) with confidence intervals, effect sizes, degrees of freedom and *P* value noted<br>*Give P values as exact values whenever suitable.*
☒	☐	For Bayesian analysis, information on the choice of priors and Markov chain Monte Carlo settings
☒	☐	For hierarchical and complex designs, identification of the appropriate level for tests and full reporting of outcomes
☒	☐	Estimates of effect sizes (e.g. Cohen's *d*, Pearson's *r*), indicating how they were calculated

Our web collection on statistics for biologists contains articles on many of the points above.

Software and code

Policy information about availability of computer code

Data collection	Metabolomics data were collected with Waters ACQUITY Reversed Phase Ultra Performance Liquid Chromatography (RP-UPLC) coupled to a Thermo-Fisher Q Exactive Plus mass spectrometer (UPLC-MS). Annotation of data performed with REFINER MS® 10.5 (GeneData, http://www.genedata.com). RNA-seq libraries were prepared and sequenced unidirectionally (50 nucleotides read length) with a read depth of 25-50 million reads/library in Illumina sequencing platform at the EMBL Genomics Core Facility in Heidelberg.
Data analysis	Data analysis and visualization was performed with R programming (4.0.4). Code utilized for analysis of the RNAseq and metabolomics data is available at: https://github.com/ASAGlab. https://github.com/ASAGlab/Regulated-IRE1-dependent-decay-RIDD-mediated-reprograming-of-lipid-metabolism-in-cancer<br>The repository contains raw transcriptomic, metabolomic data in tabular .txt format, and 3 custom scripts:<br>- edgeR_final.R : performs the differential gene expression (DEG) analysis of the transcriptomic data<br>- Metasyx_final.R : performs analysis of Metasyx metabolomics data<br>- Beatson_final.R : performs analysis of Beatson lipidomics data<br>Dependencies: "biomaRt" (v2.50.3), "colorRamps" (v2.3), "edgeR" (v3.36.0), "ggplot2" (v3.3.5), "limma" (v3.50.1), "pheatmap" (v1.0.12), "plyr" (v1.8.6), "RColorBrewer" (v1.1-2), "reshape2" (v1.4.4), "stringr" (v1.4.0), "sva" (v3.42.0)

For manuscripts utilizing custom algorithms or software that are central to the research but not yet described in published literature, software must be made available to editors and reviewers. We strongly encourage code deposition in a community repository (e.g. GitHub). See the Nature Portfolio guidelines for submitting code & software for further information.

Data

Policy information about availability of data

All manuscripts must include a data availability statement. This statement should provide the following information, where applicable:

- Accession codes, unique identifiers, or web links for publicly available datasets
- A description of any restrictions on data availability
- For clinical datasets or third party data, please ensure that the statement adheres to our policy

All data supporting the findings of this study are available at: https://github.com/ASAGlab. https://github.com/ASAGlab/Regulated-IRE1-dependent-decay-RIDD-mediated-reprograming-of-lipid-metabolism-in-cancer or from the corresponding authors upon request. The RNA sequencing data have been deposited and are available in the Gene Expression Omnibus (GEO) public database https://www.ncbi.nlm.nih.gov/gds/ under accession number GSE176454. Metabolomics data are included in this article as excel tables in Supplementary Data. The remaining data are available within the Article, Supplementary Information and Source Data file. Source data are provided with this paper.

Field-specific reporting

Please select the one below that is the best fit for your research. If you are not sure, read the appropriate sections before making your selection.

☒ Life sciences ☐ Behavioural & social sciences ☐ Ecological, evolutionary & environmental sciences

For a reference copy of the document with all sections, see nature.com/documents/nr-reporting-summary-flat.pdf

Life sciences study design

All studies must disclose on these points even when the disclosure is negative.

Sample size	Sample size was selected based on standards in the literature. All experiments were performed at least three itimes to demonstrate statistical significance.
Data exclusions	One technical repeat of the MS Metasyx experiment was excluded from the analysis due to noticeable batch effect as observed in PCA, MDS.
Replication	Each experiment was performed with at least three biological replicates. Attempts at replication were successful.
Randomization	Only biochemical or cell-based experiments (no animals) were carried out in this study and as such there was no need for randomization of our samples. The same investigator designed and performed experiments. For omics experiments, samples were analyzed in random order according to standard procedure.
Blinding	Only biochemical or cell-based experiments (no animals) were carried out in this study and as such there was no need for blinding of our samples.

Reporting for specific materials, systems and methods

We require information from authors about some types of materials, experimental systems and methods used in many studies. Here, indicate whether each material, system or method listed is relevant to your study. If you are not sure if a list item applies to your research, read the appropriate section before selecting a response.

Materials & experimental systems

n/a	Involved in the study	
☐	☒	Antibodies
☐	☒	Eukaryotic cell lines
☒	☐	Palaeontology and archaeology
☒	☐	Animals and other organisms
☒	☐	Human research participants
☒	☐	Clinical data
☒	☐	Dual use research of concern

Methods

n/a	Involved in the study	
☒	☐	ChIP-seq
☒	☐	Flow cytometry
☒	☐	MRI-based neuroimaging

Antibodies

Antibodies used	anti-IRE1 (clone 14C10) (Cell Signaling Technology, 3294, 1:1,000)<br>anti-XBP1s (clone 143F) (Biolegend, 647502, 1:1,000)<br>anti-FLAG (clone M2) (Sigma Aldrich, F1804)<br>anti-Actin (20-33) (Sigma Aldrich, A5060, 1:5,000)

	Anti-rabbit HRP-conjugated secondary antibody (Jackson Immunoresearch, 111-035-003)<br>anti-mouse HRP-conjugated secondary antibody (Jackson Immunoresearch, 115-035-003)
Validation	The antibodies used in this study were validated by the manufacturers and can be checked using their respective catalog # number on the following websites:<br>CellSignaling: https://www.cellsignal.com/about-us/cst-antibody-performance-guarantee on which there is a statement 'To ensure product performance, we validate all of our antibodies, in-house, in multiple research applications.'<br>BioLegend: ttps://www.biolegend.com/en-gb/quality/quality-control stating that each lot for purified antibodies is tested for specificity and lot-to-lot consistency.<br><br>Sigma Aldrich: https://www.sigmaaldrich.com/IE/en/products/protein-biology/antibodies/enhanced-validation-ab 'Delivering high-quality antibodies requires us to perform rigorous specificity and sensitivity testing in order to provide our customers with reliable tools that generate consistent results. We routinely perform standard validation processes across our antibody portfolio. Our standard antibody validation processes include verification for each recommended immunodetection application. Each of the thousands of antibodies in our portfolio are certified through our standard validation process to ensure quality and reproducibility.'

Eukaryotic cell lines

Policy information about cell lines

Cell line source(s)	MDA-MB-231, MDA-MB-468, MCFl0A, HCC1806, BT549 and HEK293T were received from ATCC
Authentication	MDA-MB231 cells were STR profiled in October 2017. MCF10A cells were STR profiled in July 2021.
Mycoplasma contamination	Cells were tested once per month and were negative.
Commonly misidentified lines<br>(See ICLAC register)	No
